# Mitochondrial phosphatidylethanolamine modulates UCP1 to promote brown adipose thermogenesis

**DOI:** 10.1126/sciadv.ade7864

**Published:** 2023-02-24

**Authors:** Jordan M. Johnson, Alek D. Peterlin, Enrique Balderas, Elahu G. Sustarsic, J. Alan Maschek, Marisa J. Lang, Alejandro Jara-Ramos, Vanja Panic, Jeffrey T. Morgan, Claudio J. Villanueva, Alejandro Sanchez, Jared Rutter, Irfan J. Lodhi, James E. Cox, Kelsey H. Fisher-Wellman, Dipayan Chaudhuri, Zachary Gerhart-Hines, Katsuhiko Funai

**Affiliations:** ^1^ Diabetes and Metabolism Research Center, University of Utah, Salt Lake City, UT, USA.; ^2^ Department of Nutrition and Integrative Physiology, University of Utah, Salt Lake City, UT, USA.; ^3^ Utah Center for Clinical and Translational Research, University of Utah, Salt Lake City, UT, USA.; ^4^ Nora Eccles Harrison Cardiovascular Research and Training Institute, Division of Cardiovascular Medicine, Department of Internal Medicine, University of Utah, Salt Lake City, UT, USA.; ^5^ Novo Nordisk Foundation Center for Basic Metabolic Research, University of Copenhagen, Copenhagen, Denmark.; ^6^ Metabolomics Core Research Facility, University of Utah, Salt Lake City, UT, USA.; ^7^ Department of Biochemistry, University of Utah, Salt Lake City, UT, USA.; ^8^ Howard Hughes Medical Institute, University of Utah, Salt Lake City, UT, USA.; ^9^ Department of Integrative Biology and Physiology, University of California Los Angeles, Los Angeles, CA, USA.; ^10^ Division of Urology, Department of Surgery, Huntsman Cancer Institute, University of Utah, Salt Lake City, UT, USA.; ^11^ Division of Endocrinology, Metabolism and Lipid Research, Washington University School of Medicine, St. Louis, MO, USA.; ^12^ East Carolina Diabetes and Obesity Institute, East Carolina University, Greenville, NC, USA.; ^13^ Molecular Medicine Program, University of Utah, Salt Lake City, UT, USA.

## Abstract

Thermogenesis by uncoupling protein 1 (UCP1) is one of the primary mechanisms by which brown adipose tissue (BAT) increases energy expenditure. UCP1 resides in the inner mitochondrial membrane (IMM), where it dissipates membrane potential independent of adenosine triphosphate (ATP) synthase. Here, we provide evidence that phosphatidylethanolamine (PE) modulates UCP1-dependent proton conductance across the IMM to modulate thermogenesis. Mitochondrial lipidomic analyses revealed PE as a signature molecule whose abundance bidirectionally responds to changes in thermogenic burden. Reduction in mitochondrial PE by deletion of phosphatidylserine decarboxylase (PSD) made mice cold intolerant and insensitive to β3 adrenergic receptor agonist–induced increase in whole-body oxygen consumption. High-resolution respirometry and fluorometry of BAT mitochondria showed that loss of mitochondrial PE specifically lowers UCP1-dependent respiration without compromising electron transfer efficiency or ATP synthesis. These findings were confirmed by a reduction in UCP1 proton current in PE-deficient mitoplasts. Thus, PE performs a previously unknown role as a temperature-responsive rheostat that regulates UCP1-dependent thermogenesis.

## Teaser

Phosphatidylethanolamine performs a previously unknown role in brown adipose thermogenesis by modulating UCP1 activity.

## INTRODUCTION

Nearly two-thirds of adults in the United States are overweight or obese, putting them at high risk of developing cardiovascular disease, type 2 diabetes, and cancer (*1*, *2*). Increasing thermogenesis in adipose tissue is a potential means of increasing energy expenditure and preventing hyperglycemia. Uncoupling protein 1 (UCP1), an integral membrane protein that resides in the inner mitochondrial membrane (IMM), is largely responsible for thermogenesis in brown and beige adipocytes. UCP1 promotes inefficient oxidative phosphorylation (OXPHOS) by dissipating the IMM proton gradient independent of adenosine triphosphate (ATP) synthesis. Overexpression of UCP1 in brown and white adipose tissue increases energy expenditure and may prevent diet-induced obesity in mice (*3*).

In both mice and humans, UCP1-positive brown and/or beige adipocytes are primarily activated by changes in environmental temperature. Evidence suggests that IMM lipid composition adapts to cellular energetic demand (*4*). Thus, we hypothesized that temperature can influence the lipid composition of IMM to regulate UCP1 function. The IMM is a protein-rich phospholipid bilayer, whose lipid composition can substantially affect enzyme activity (*5*). Cone-shaped phosphatidylethanolamine (PE) and cardiolipin (CL) are particularly enriched in cristae, where they are thought to create the negative curvature and support the function of the complexes of the electron transport system (ETS) (*6*–*8*). Mutations in the genes of mitochondrial PE or CL biosynthesis are known to cause severe mitochondrial disease, characterized by poor cristae formation, impaired capacity for OXPHOS, blunted efficiency of electron transfer, reduced ETS complex activity, and decreased supercomplex assembly (*9*–*14*). CL and PE abundances both increase with cold exposure, and CL is required for thermogenesis partly via the transcriptional regulation of UCP1 (*15*, *16*). However, it is unknown whether these lipids directly regulate UCP1 activity to modulate thermogenesis.

Here, we sought to determine whether interventions that activate brown adipose thermogenesis were associated with distinct phospholipid signatures in mitochondria. Comprehensive mitochondrial lipidomic analyses of brown adipose tissue (BAT) suggested that PE, not CL, is the primary lipid class responsive to altering energy demand in BAT. Genetic ablation of mitochondrial PE biosynthesis, but not CL, reduced UCP1-dependent respiration. Further, direct patch-clamp measurements of mitoplasts synthesized from these mitochondria showed that reduction in PE lowered UCP1 proton current. These findings implicate mitochondrial PE as an energy-responsive IMM element that regulates UCP1 activity and BAT thermogenesis.

## RESULTS

### PE is the energy-responsive phospholipid in BAT mitochondria

**Fig. 1.**
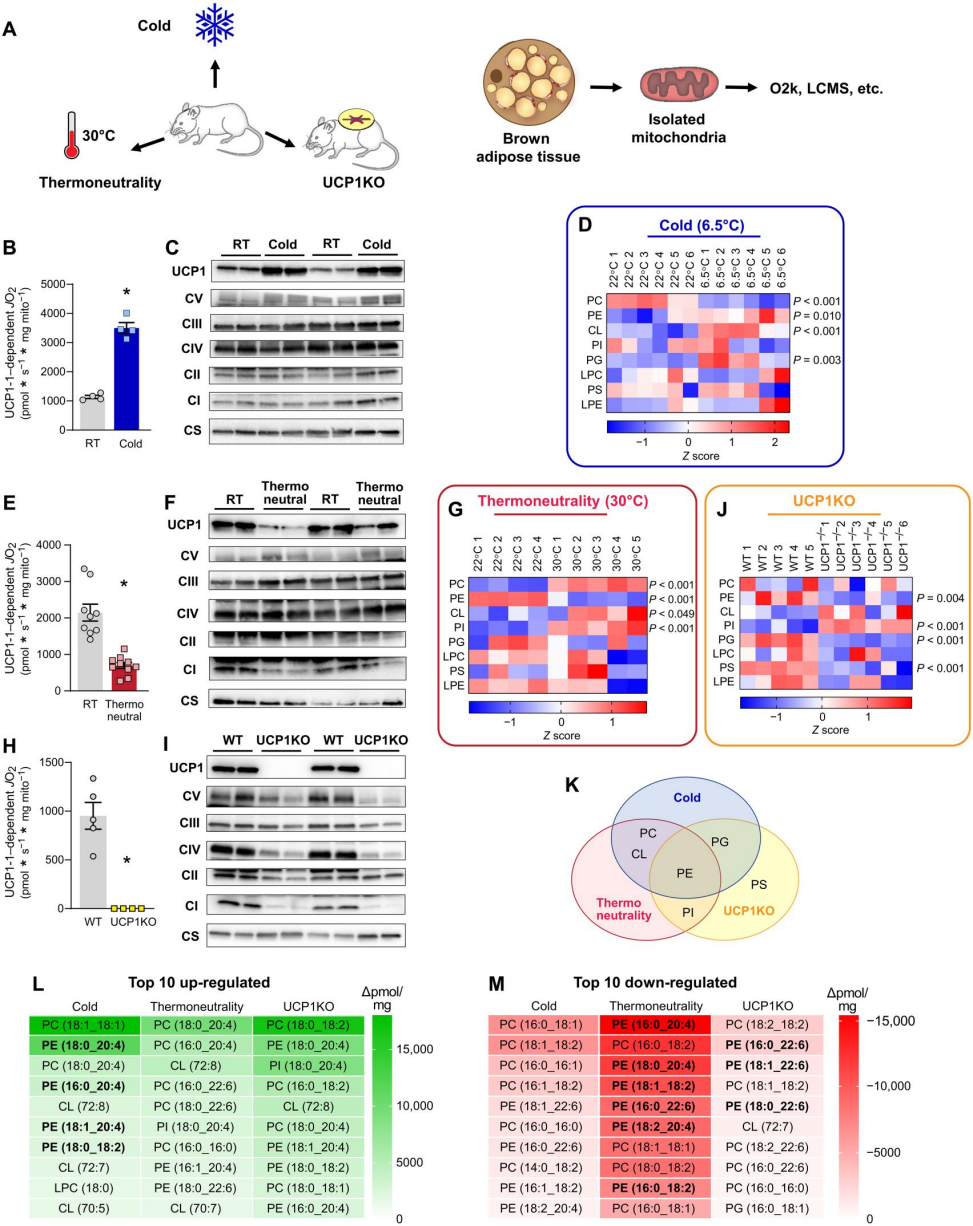
Brown adipose mitochondrial lipidome is highly responsive to changes in thermogenic burden. (**A**) Experimental design to assess the influences of cold, thermoneutrality, or UCP1 knockout on BAT mitochondrial energetics and lipidome. (**B**) UCP1-dependent respiration stimulated by malate and pyruvate in BAT mitochondria from C57BL/6J mice housed at RT or 6.5°C for 7 days. *n* = 4 per group. (**C**) Protein abundance of UCP1, ETS subunits, and citrate synthase in BAT mitochondria isolated from C57BL/6J mice housed at RT or 6.5°C for 7 days. (**D**) Summary heatmap of changes in BAT mitochondrial phospholipidome in C57BL/6J mice housed at RT or 6.5°C for 7 days. Abundance of lipids in each lipid class was derived from individual lipid species in fig. S1. *n* = 6 per group. (**E**) UCP1-dependent respiration in BAT mitochondria from C57BL/6J mice housed at RT or 30°C for 30 days. *n* = 9 to 10 per group. (**F**) Protein abundance of UCP1, ETS subunits, and citrate synthase in BAT mitochondria from C57BL/6J mice housed at RT or 30°C for 30 days. (**G**) Summary heatmap of changes in BAT mitochondrial phospholipidome in C57BL/6J mice housed at RT or 30°C for 30 days. Abundance of lipids in each lipid class was derived from individual lipid species in fig. S2. *n* = 4 to 5 per group. (**H**) UCP1-dependent respiration in BAT mitochondria from wild-type (WT) and UCP1KO mice. *n* = 4 to 5 per group. (**I**) Protein abundance of UCP1, ETS subunits, and citrate synthase in BAT mitochondria from WT and UCP1KO mice. (**J**) Summary heatmap of changes in BAT mitochondrial phospholipidome in WT and UCP1KO mice. *n* = 5 to 6 per group. (**K**) Venn diagram demonstrating PE as the only class of lipids that is influenced by cold, thermoneutrality, and UCP1 knockout. (**L**) A list of the top 10 mitochondrial lipid species that are up-regulated with cold, thermoneutrality, or UCP1 knockout. (**M**) A list of the top 10 mitochondrial lipid species that are down-regulated with cold, thermoneutrality, or UCP1 knockout. Data are presented as ±SEM. *P < 0.05.

Interventions that influence energy balance such as diet, exercise, and ambient temperature are known to alter mitochondrial lipid composition in a variety of tissues (*4*, *17*–*21*). We set out to determine whether interventions that alter the energic burden of BAT thermogenesis coincide with changes in the mitochondrial lipid milieu (Fig. 1A). We first compared wild-type C57BL/6J mice that were housed at 6.5°C (cold) for 1 week to 22°C room temperature (RT)–housed controls. Deep phenotyping of BAT mitochondria from these mice indicated markedly elevated respiration (fig. S1A) with a modest reduction in the rate of ATP synthesis in the cold-housed mice (fig. S1B). The reduced efficiency for OXPHOS (ATP/O; fig. S1C) was not due to increased electron leak (fig. S1D) but because of a robust increase in UCP1-dependent respiration (Fig. 1B; quantified by subtracting *J*O_2_ with UCP1 inhibition from total *J*O_2_). Consistent with these findings, the increase in mitochondrial respiration occurred concomitant with increased abundance of UCP1 in mitochondria, without changes in the quantity of ETS enzymes (Fig. 1C).

Comprehensive lipid mass spectrometric analyses of BAT mitochondria revealed significant increases in PE, CL, and phosphatidylglycerol (PG) and a decrease in phosphatidylcholine (PC) (Fig. 1D and fig. S1, E to L). Cold-stimulated increase in CL and PG was consistent with our previous report (*15*). We also observed an equally robust increase in mitochondrial PE, analogous to exercise-induced increase in mitochondrial PE that was observed in skeletal muscle (*4*). The changes in these lipids coincided with increased expression of the biosynthetic enzymes responsible for mitochondrial PE and CL production (fig. S1M).

Next, we examined BAT mitochondria from wild-type C57BL/6J mice that were housed at 30°C (thermoneutrality) or 22°C (RT) for 4 weeks. Mice exhibit a modestly elevated BAT-mediated thermogenesis at RT (~22°C) compared to thermoneutrality (*22*, *23*). As expected, mitochondrial phenotyping experiments showed reduced respiration (fig. S2A) and increased capacity for ATP synthesis in the thermoneutral-housed mice compared to RT-housed mice (fig. S2B). The increased efficiency of OXPHOS under thermoneutral conditions (fig. S2C) was not due to altered electron leak (fig. S2D) but because of markedly diminished UCP1-dependent respiration (Fig. 1E). Reduced UCP1 content in thermoneutral mitochondria was consistent with the diminished UCP1-dependent *J*O_2_ (Fig. 1F). Thermoneutrality influenced the BAT mitochondrial lipidome in some unexpected ways (Fig. 1G and fig. S2, E to M). Abundance of CL was higher, not lower, in mice housed at thermoneutrality compared to mice housed at RT. Instead, PE was the only lipid class whose abundance was down-regulated with thermoneutrality. In addition to CL, PC and phosphatidylinositol (PI) were also increased with thermoneutrality.

To further show that PE, not CL, is the energy-responsive lipid in the IMM, we also examined UCP1 null (UCP1KO) mice. BAT mitochondria from UCP1KO mice are similar to mitochondria from thermoneutral conditions in that they lack UCP1-driven thermogenesis. UCP1 deletion reduced respiration (fig. S3A) and increased efficiency of OXPHOS (fig. S3, B and C) that was due to a lack of UCP1-dependent respiration (Fig. 1, H and I, and fig. S3D). Phospholipidomic analyses revealed changes in the mitochondrial lipid milieu with UCP1 deletion that were remarkably similar to what was observed in changes with thermoneutrality (Fig. 1J and fig. S3, E to M). CL was not responsive to UCP1 deletion (trending for an increase rather than a decrease). Instead, PE, PG, and PS were lower, while PI was higher in UCP1KO mice compared to wild-type controls. Together, PE was the only energy-responsive lipids across the three models that were examined (Fig. 1K). PE is highly abundant (40% of total BAT mitochondrial phospholipids) and is important for OXPHOS (*4*, *9*, *24*). PE ranked consistently high among the lipid species whose abundance was up-regulated with cold and down-regulated with thermoneutrality or UCP1 knockout when compared to relevant control samples (Fig. 1, L and M). On the basis of these observations, we hypothesized that PE was a likely candidate to regulate UCP1 function to modulate thermogenesis. Nevertheless, on the basis of our previous findings that CL is an important regulator of BAT thermogenesis, we further examined the role of both PE and CL in UCP1-dependent thermogenesis.

### BAT-specific deletion of CL synthase impairs thermogenesis

**Fig. 2.**
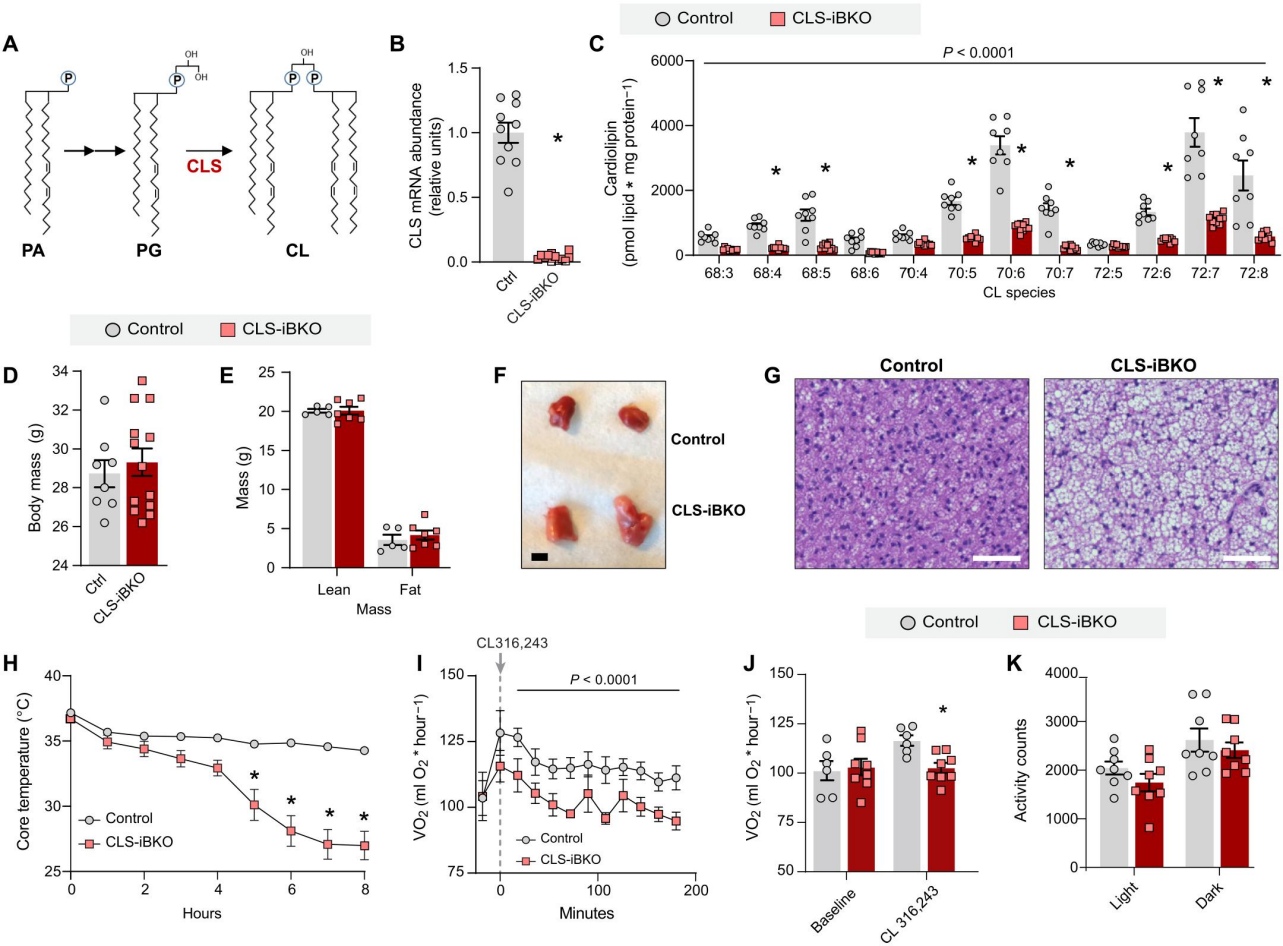
Loss of mitochondrial CL impairs brown adipose thermogenesis. (**A**) Schematic for mitochondrial CL biosynthesis. (**B**) CLS mRNA abundance in BAT from control and CLS-iBKO mice. *n* = 10 to 12 per group. (**C**) Abundance of mitochondrial CL species in BAT from control and CLS-iBKO mice. *n* = 8 to 10 per group. (**D**) Body mass. *n* = 8 to 13 per group. (**E**) Body composition. *n* = 5 to 7 per group. (**F**) Representative intrascapular BAT depots from control and CLS-iBKO mice. (**G**) Hematoxylin and eosin staining. Scale bars, 50 μM. (**H**) Core body temperature of mice subjected to an acute cold tolerance test at 4°C. *n* = 7 to 9 per group. (**I**) Time course of whole-body oxygen consumption in mice before and after injection of CL-316,243. *n* = 6 to 8 per group. (**J**) Mean whole-body oxygen consumption following administration of CL-316,243. *n* = 6 to 8 per group. (**K**) Spontaneous movement in metabolic cages. *n* = 8 per group. Data are presented as ±SEM. *P < 0.05.

We previously demonstrated that deletion of CL synthase (CLS) (Fig. 2A) driven by adiponectin-Cre, tamoxifen-inducible Rosa26-Cre, and tamoxifen-inducible UCP1-Cre (UCP1 Cre-ERT2) impairs thermogenesis and leads to insulin resistance (*15*). The majority of these experiments were performed in adiponectin-Cre–driven CLS knockout mice. Here, we performed additional studies on CLS knockout driven by the inducible UCP1-Cre (CLS-iBKO) to conduct a more in-depth mitochondrial phenotyping.

As expected, the CLS mRNA abundance was reduced in CLS-iBKO mice compared to control mice (Fig. 2B). However, because of its relatively slow turnover, CL levels were not substantially reduced until 4 weeks after tamoxifen injection (Fig. 2C). Thus, we performed all subsequent analyses of the CLS-iBKO and control mice at 4 weeks after tamoxifen injection. Consistent with CLS’s activity of synthesizing CL from PG, CLS deletion also increased mitochondrial PG compared to controls (fig. S4A). CLS-iBKO and control mice did not differ in body mass, lean mass, or fat mass (Fig. 2, D and E), but BAT was both larger and paler in CLS-iBKO mice (Fig. 2F and fig. S4B). Histological sections of BAT revealed increased lipid accumulation in the CL-deficient samples (Fig. 2G), potentially suggesting impaired thermogenic capacity. Reduced CL levels impaired cold tolerance when mice were challenged at 4°C (Fig. 2H and fig. S4C). Furthermore, although administration of the β3 agonist CL-316,243 increased whole-body oxygen consumption (VO_2_) in control mice, CLS-iBKO mice were refractory to CL-316,243–induced changes in VO_2_, which indicates defective thermogenesis (Fig. 2, I and J). Twenty-four–hour calorimetry at RT showed no effect of CLS deletion on VO_2_, respiratory exchange ratio (RER), and activity, suggesting that these animals are otherwise not hypoenergetic (Fig. 2K and fig. S4, D and E).

### CLS deletion impairs thermogenesis independent of its effect on UCP1-dependent respiration

**Fig. 3.**
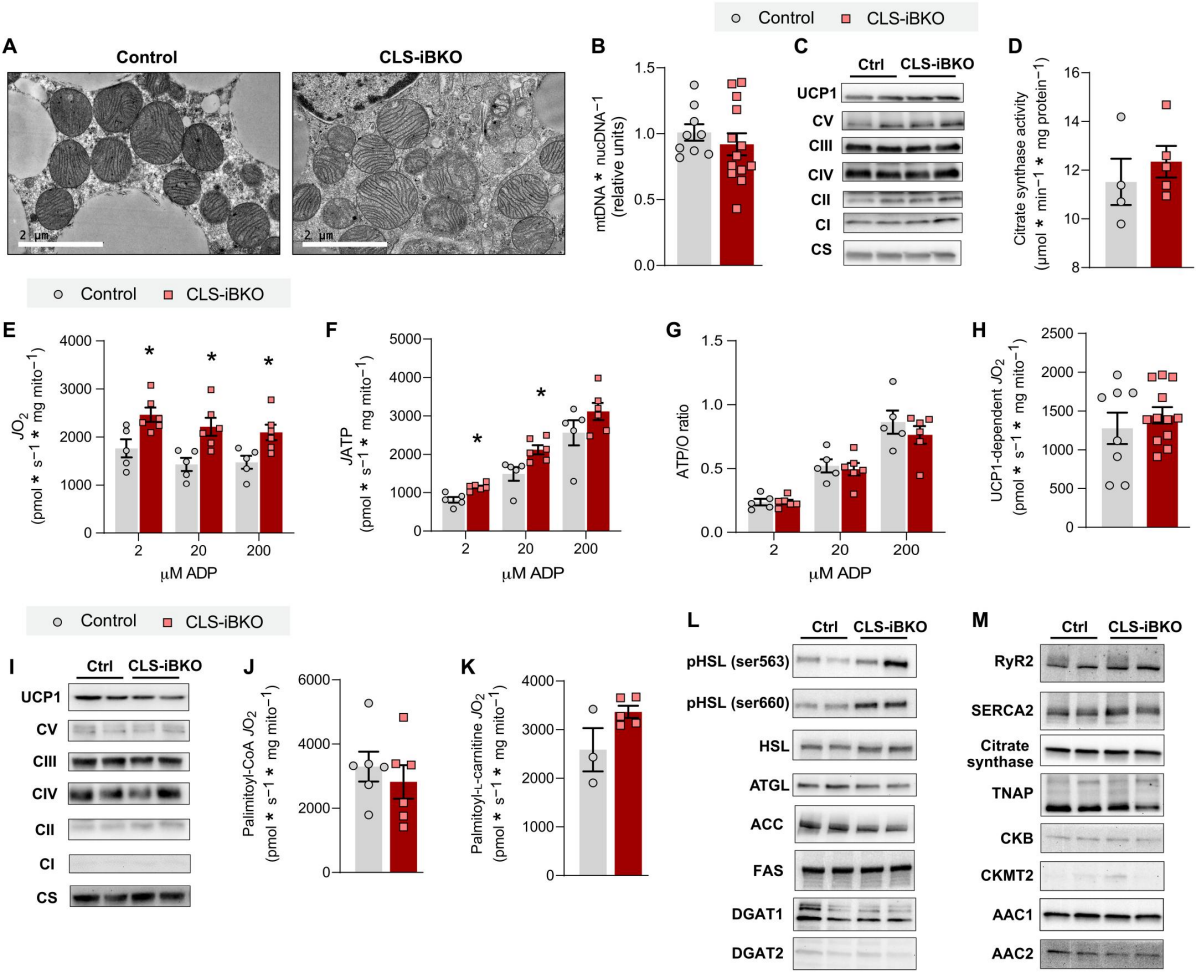
CL deficiency does not impair capacity for uncoupled respiration. (**A**) Transmission electron microscopy images of BAT mitochondria from control and CLS-iBKO mice. Scale bars, 2 μM. (**B**) mtDNA levels normalized to nucDNA in BAT from control and CLS-iBKO mice. *n* = 9 to 13 per group. (**C**) Protein abundance of UCP1, ETS subunits, and CS in whole BAT homogenates. (**D**) CS activity in whole BAT homogenates. *n* = 4 to 5 per group. (**E**) Mitochondrial O_2_ consumption (*J*O_2_) in BAT mitochondria from control and CLS-iBKO mice, measured in the presence of 5 mM pyruvate, 0.2 mM malate, 5 mM glutamate, 5 mM succinate, and 2, 20, and 200 μM ADP. *n* = 5 to 6 per group. (**F**) ATP production (*J*ATP) in the presence of 5 mM pyruvate, 0.2 mM malate, 5 mM glutamate, 5 mM succinate, and 2, 20, and 200 μM ADP. *n* = 5 to 6 per group. (**G**) Mitochondrial coupling efficiency (ATP/O ratio). *n* = 5 to 6 per group. (**H**) UCP1-dependent respiration in BAT mitochondria from control and CLS-iBKO mice. Respiration was stimulated by 5 mM pyruvate and 0.2 mM malate, and UCP1 was subsequently inhibited by 4 mM guanosine diphosphate (GDP). *n* = 8 to 12 per group. (**I**) Protein abundance of UCP1, ETS subunits, and CS in isolated mitochondria. (**J**) UCP1-dependent respiration stimulated by 0.2 mM malate, 5 mM carnitine, and 20 μM palmitoyl-CoA. *n* = 6 per group. (**K**) UCP1-dependent respiration stimulated by 0.2 mM malate, 5 mM carnitine, and 20 μM palmitoyl-l-carnitine. *n* = 3 to 5 per group. (**L**) Abundance of phosphorylated (Ser^563^ and Ser^660^) and total hormone-sensitive lipase (HSL), adipose triglyceride lipase (ATGL), acetyl-CoA carboxylase (ACC), fatty acid synthase (FAS), and diacylglycerol acyltransferase 1 and 2 (DGAT1 and DGAT2) in whole BAT homogenates. (**M**) Abundance of ryanodine receptor 2 (RyR2), sarco/endoplasmic reticulum Ca^2+^–adenosine triphosphatase 2 (SERCA2), tissue nonspecific alkaline phosphatase (TNAP), creatine kinase B (CKB), creatine kinase, mitochondrial 2 (CKMT2), and ADP/ATP carrier 1 and 2 (AAC1 and AAC2) in whole BAT homogenates. Data are presented as ±SEM. *P < 0.05.

On the basis of CL’s localization to the IMM and its effect on OXPHOS, we hypothesized that CLS deletion lowers CL to reduce UCP1 activity. To test this, we phenotyped BAT mitochondria from control and CLS-iBKO mice. Electron micrograph of BAT unexpectedly revealed no substantial deformities in mitochondrial shape or cristae density in CLS-iBKO–derived samples compared to wild-type–derived samples (Fig. 3A). Quantification of mitochondrial density by mitochondrial DNA (mtDNA)/nuclear DNA (nucDNA) (Fig. 3B), protein quantifications of UCP1, complex I-V, and citrate synthase (Fig. 3C), and citrate synthase activity (Fig. 3D) showed no difference between BAT from control and CLS-iBKO mice. Bioenergetic experiments further revealed unexpected effects of CLS deletion on mitochondria. Adenosine diphosphate (ADP)–dependent respiration was elevated, not reduced, in CLS-lacking mitochondria (Fig. 3E). The increased respiration coincided with an increased rate of ATP synthesis (Fig. 3F) and no overall effect on the ATP/O ratio (Fig. 3G) or electron leak (fig. S4F). Notably, in stark contrast to our hypothesis, CLS deletion had no effect on UCP1-dependent respiration (Fig. 3H).

These findings indicate that, to a substantial extent, CL is dispensable for UCP1 activity. Nevertheless, it is important to acknowledge that CL levels did not reach zero in our CLS-iBKO mice, which leaves open the distinct possibility that the residual CL is sufficient to maintain UCP1 activity in BAT mitochondria from CLS-iBKO mice. We attempted alternative approaches to diminish CL further, but we were unable to achieve this by genetically reducing CLS alone. First, we performed a time-course experiment to see whether prolonging terminal experiments would allow CL levels to be more completely depleted. However, new adipocytes began to emerge between 6 and 8 weeks after tamoxifen injection, preventing mitochondrial CL from reaching zero. We also attempted to reduce mitochondrial CL levels to zero in vitro by culturing primary brown adipocytes. However, deleting CLS in preadipocytes prevented brown adipocyte differentiation, and deleting CLS after differentiation did not lower mitochondrial CL beyond the level that was achievable in vivo. Thus, we were unable to conclude that CL is completely dispensable for UCP1 activity. Nevertheless, it is important to point out that CLS-iBKO mice exhibited cold intolerance and reduced VO_2_ induced by β3 agonist (Fig. 2, H to J) despite incomplete removal of CL. Therefore, it can be concluded that defective thermogenesis in CLS-iBKO mice is not exclusively due to the direct effect of CL on UCP1. In turn, our findings suggest that CLS influences thermogenesis independent of its action on UCP1, including the possibility that CLS may have an alternate enzyme activity in addition to the synthesis of CL.

How does CLS deletion impair thermogenesis independent of modulating UCP1 activity? Control and CLS-iBKO mice did not differ in UCP1, complex I-V, or citrate synthase content per unit of mitochondria (Fig. 3I). Combined with unchanged total cellular UCP1 content (Fig. 3C), these data show that reduced thermogenesis in CLS-iBKO mice is not due to CLS regulating the UCP1 protein level. We then examined the possibility that CLS affects the ability of fatty acids to activate UCP1. However, respiration driven by palmitoyl–coenzyme A (CoA) was not different between the groups (Fig. 3J). On the basis of the possibility that low CL may interfere with the transport of fatty acids across the outer mitochondrial membrane (OMM) and IMM (*25*, *26*), we also tested respiration induced by palmitoyl-l-carnitine. However, similar to respiration driven by palmitoyl-CoA, CLS deletion had no effect on respiration driven by palmitoyl-l-carnitine (Fig. 3K). These data suggest that CL deletion does not interfere with the ability of fatty acids to activate UCP1, nor does it affect the ability of the carnitine system to transport fatty acids across mitochondrial membranes. We also examined the possibility that CL influences the availability of fatty acids necessary to activate UCP1. BAT thermogenesis is activated in vivo by noradrenaline binding to the β3 G protein–coupled receptor (GPCR) on brown adipocytes. The signaling cascade activates hormone-sensitive lipase (HSL) and adipose triglyceride lipase (ATGL), both of which are required to cleave triacylglycerol into individual free fatty acids (*27*, *28*). Deletion of CLS did not lower the protein abundance of either HSL or ATGL nor affect two activating phosphorylation sites on HSL (Fig. 3L) (*29*–*31*). We also examined enzymes of lipogenesis, considering that lipogenesis and lipolysis may constitute a futile cycle (*32*, *33*), but we detected no differences for diacylglycerol acyltransferase (DGAT), fatty acid synthase (FAS), or acetyl-CoA carboxylase (ACC) (Fig. 3L). Last, we also examined other known energy-dissipating systems such as endoplasmic reticulum calcium cycling [ryanodine receptor 2 (RyR2) and sarco/endoplasmic reticulum Ca^2+^–adenosine triphosphatase 2 (SERCA2)] (*34*), creatine cycling [creatine kinase B (CKB), tissue-nonspecific alkaline phosphatase (TNAP), and creatine kinase, mitochondrial 2 (CKMT2)] (*35*), and ADP/ATP carrier (AAC1 and AAC2) (*36*) and found that CLS deletion does not affect abundance of these enzymes (Fig. 3M). In summary, the role of CLS in regulating BAT thermogenesis is independent of fatty acid–mediated UCP1 activation, or obvious changes in the protein abundance of enzymes involved other known futile cycling mechanisms.

### Mitochondrial PE is essential for brown adipose thermogenesis

**Fig. 4.**
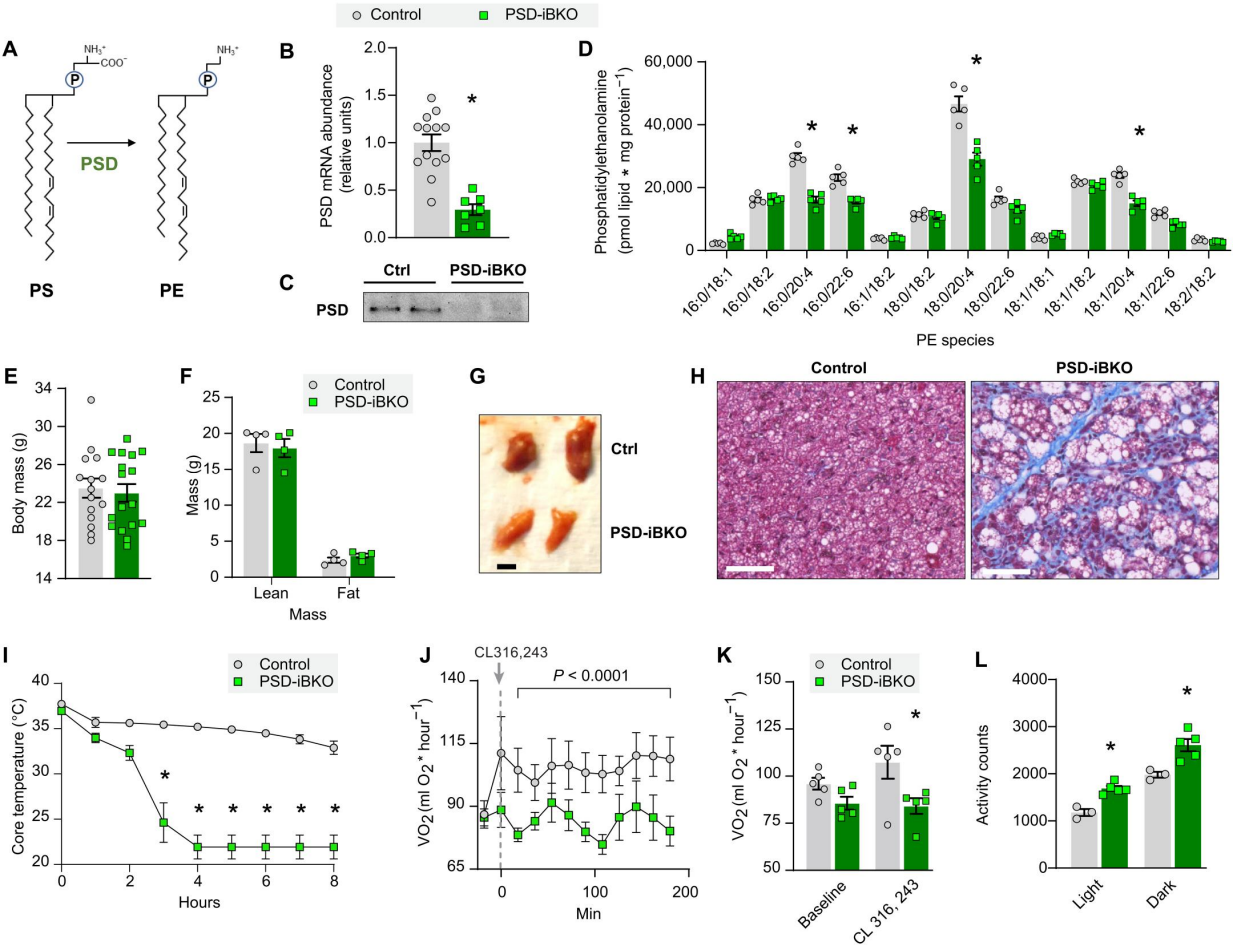
Loss of mitochondrial PE impairs brown adipose thermogenesis. (**A**) Schematic for mitochondrial PE biosynthesis. (**B**) PSD mRNA abundance in BAT from control and PSD-iBKO mice. *n* = 7 to 13 per group. (**C**) Representative images of PSD Western blot in BAT from control and PSD-iBKO mice. (**D**) Mitochondrial PE species abundance in BAT 2 weeks after tamoxifen injection. *n* = 5 per group. (**E**) Body mass. *n* = 15 to 17 per group. (**F**) Body composition. *n* = 4 per group. (**G**) Representative intrascapular BAT depots from control and PSD-iBKO mice. (**H**) Masson’s trichrome blue staining of BAT. Scale bars, 50 μM. (**I**) Core body temperature of mice subjected to an acute cold tolerance test at 4°C. *n* = 7 to 8 per group. (**J**) Time course of whole-body oxygen consumption in mice before and after injection of CL-316,243. *n* = 5 per group. (**K**) Mean whole-body oxygen consumption following administration of CL-316,243. *n* = 5 per group. (**L**) Spontaneous movement in metabolic cages. *n* = 3 to 5 per group. Data are presented as ±SEM. *P < 0.05.

Mitochondria have the intrinsic ability to modulate the abundance of PE by an IMM-resident enzyme phosphatidylserine decarboxylase (PSD) (Fig. 4A) (*4*, *37*, *38*). A mitochondrial importer for PE is not known to exist, and stable isotope studies suggest that PE is not imported into mitochondria (*39*). Thus, mitochondrial PE is likely almost exclusively generated by PSD. Because mitochondrial PE was the most affected class of lipids that were energy responsive in BAT, we developed a system whereby we could manipulate the levels of mitochondrial PE and test its role in BAT thermogenesis. To this end, we generated mice with tamoxifen-inducible UCP1-Cre–driven knockout of PSD (PSD-iBKO) to block mitochondrial PE synthesis (Fig. 4, B and C).

A recent report suggests that constitutive UCP1-Cre mice have leaky Cre expression in other key metabolic tissues such as the hypothalamus and kidney resulting in nonspecific genetic recombination (*40*). We examined whether the tamoxifen-inducible UCP1-Cre (UCP1 Cre-ERT2) we used resulted in PSD knockout in other tissues. Although we did detect a small level of Cre expression in the hypothalamus and some other nonadipose tissues, PSD expression was not significantly altered in any tissue but BAT (fig. S5, A and B). These results suggest that any observed mitochondrial or thermogenic phenotypes in PSD-iBKO mice are unlikely to result from changes in PSD expression in cell types other than brown or beige adipocytes.

Unlike CLS-iBKO mice, which were studied 4 weeks after tamoxifen injection, levels of mitochondrial PE were reduced 2 weeks after tamoxifen injection in PSD-iBKO mice (Fig. 4D), demonstrating a more rapid turnover of PE than CL. Extending the terminal experiments to 4 weeks after tamoxifen injection did not further lower mitochondrial PE level (fig. S6A), likely due to adipocyte turnover and compensatory PE synthesis by mitochondrial lyso-PE acylation (Lands cycle) (fig. S6B). There were also changes in the levels of mitochondrial PS (the substrate of PSD), which further demonstrated loss of PSD activity (fig. S6C). Thus, we performed all our subsequent experiments on PSD-iBKO mice 2 to 4 weeks after tamoxifen injection.

Reduction of PSD driven by UCP1 Cre-ERT2 did not appear to have an overt phenotype in an unstressed condition; for instance, control and PSD-iBKO mice did not differ in body mass or body composition (Fig. 4, E and F). At the tissue level, however, BAT from PSD-iBKO mice were smaller and paler compared to control mice (Fig. 4G and fig. S6D). This was in contrast to BAT from CLS-iBKO mice that were larger compared to their controls. Histological analyses revealed larger lipid droplets and fibrosis in BAT from PSD-iBKO mice (Fig. 4H). Intracellular lipid accumulation may suggest reduced thermogenic capacity (*41*, *42*). PSD-iBKO mice demonstrated substantial reduction in cold tolerance compared to controls (Fig. 4I and fig. S6E), displaying an even more intolerant phenotype than CLS-iBKO mice (fig. S6F). Furthermore, PSD-iBKO mice were not responsive to CL-316,243–induced increase whole-body oxygen consumption (Fig. 4, J and K), suggesting impaired BAT thermogenesis. Potentially as a mechanism to compensate for the lack of brown adipose thermogenesis, PSD-iBKO mice were more active than control mice during light and dark cycles (Fig. 4L). This coincided with trends for elevated whole-body oxygen consumption and RER, with PSD deletion (fig. S6, G and H).

### Mitochondrial PE is required for proton flux through UCP1

**Fig. 5.**
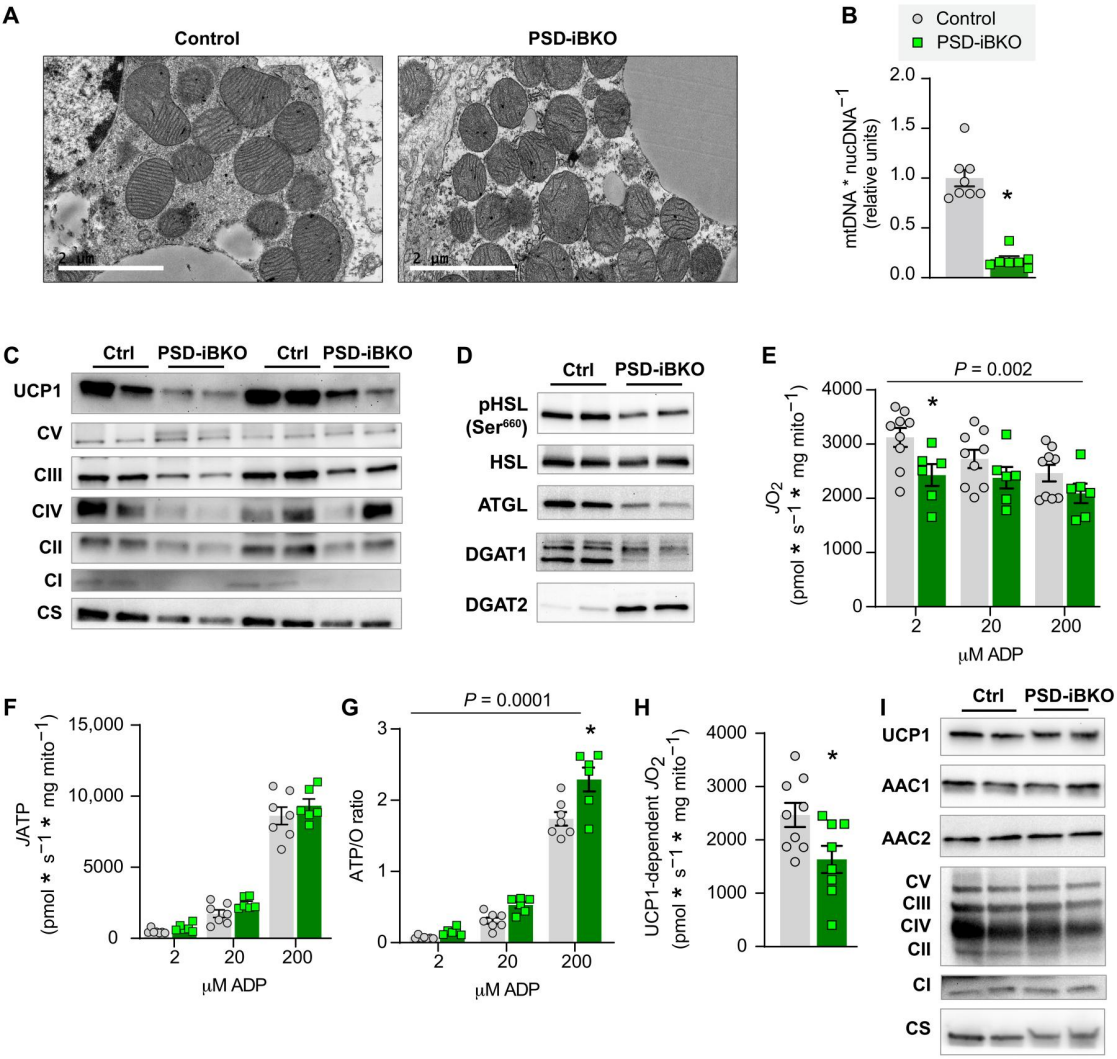
Mitochondrial PE is essential for UCP1-dependent respiration. (**A**) Transmission electron microscopy images of BAT mitochondria from control and PSD-iBKO mice. Scale bars, 2 μM. (**B**) mtDNA levels normalized to nucDNA in BAT from control and PSD-iBKO mice. *n* = 7 to 8 per group. (**C**) Protein abundance of UCP1, ETS subunits, and CS in whole BAT homogenates. (**D**) Abundance of phosphorylated (Ser^660^) and total HSL, ATGL, DGAT1, and DGAT2 in whole BAT homogenates. (**E**) Mitochondrial O_2_ consumption in BAT mitochondria from control and PSD-iBKO mice, measured in the presence of 5 mM pyruvate, 0.2 mM malate, 5 mM glutamate, 5 mM succinate, and 2, 20, and 200 μM ADP. *n* = 8 to 9 per group. (**F**) ATP production in the presence of 5 mM pyruvate, 0.2 mM malate, 5 mM glutamate, 5 mM succinate, and 2, 20, and 200 μM ADP. *n* = 6 to 7 per group. (**G**) Mitochondrial coupling efficiency (ATP/O ratio). *n* = 6 to 7 per group. (**H**) UCP1-dependent respiration in BAT mitochondria from control and PSD-iBKO mice. Respiration was stimulated by 5 mM pyruvate and 0.2 mM malate, and UCP1 was subsequently inhibited by 4 mM GDP. *n* = 8 to 9 per group. (**I**) Protein abundance of UCP1, AAC1, AAC2, CS, and ETS subunits in isolated mitochondria. Data are presented as ±SEM. *P < 0.05.

To investigate the mechanistic role of mitochondrial PE in thermogenesis, we examined BAT mitochondria from control and PSD-iBKO mice. Electron micrograph revealed robust disorganization of IMM, particularly with decreased density of cristae (Fig. 5A). Quantification of mtDNA/nucDNA or mitochondrial enzymes by Western blotting revealed that PSD deletion substantially lowered mitochondrial density per cell (Fig. 5, B and C). The reduction in mitochondrial content and poor cristae formation likely directly contribute to reduced thermogenic capacity in BAT. We also found that PSD deletion promoted decreases in phosphorylated HSL, total ATGL, and total DGAT1 and a marked increase in total DGAT2 (Fig. 5D). We believe that these changes in lipogenesis and lipolysis occurred in response to a robust reduction in BAT thermogenic capacity. It is noteworthy that DGAT2, but not DGAT1, is associated with lipid droplet recruitment to mitochondria and the formation of peridroplet mitochondria (*43*, *44*).

Cristae misalignment suggests that PSD deletion also lowered the oxidative capacity per unit of mitochondria. High-resolution respirometry and fluorometry of isolated mitochondria from BAT revealed reduced ADP-dependent respiration in PSD-iBKO mice compared to controls (Fig. 5E). Notably, the reduction was not due to changes in the rate of ATP synthesis (Fig. 5F) or electron leak (fig. S6F). Instead, increased ATP/O ratio (Fig. 5G) coincided with a robust reduction in UCP1-dependent respiration (Fig. 5H). The reduction in UCP1-dependent respiration occurred in the absence of changes in UCP1 protein abundance per unit of mitochondria (Fig. 5I). We also assessed the abundance of AAC1 and AAC2 in BAT mitochondria from these mice and found no difference between the groups (Fig. 5I). It is important to note that the contribution of AAC on mitochondrial proton flux is minimal compared to UCP1 in BAT (*36*). Consistent with unaltered ATP synthesis, abundances of complex I-V in mitochondria were not different between control and PSD-iBKO mice. These findings suggest that mitochondrial PE is essential for optimal UCP1 function.

**Fig. 6.**
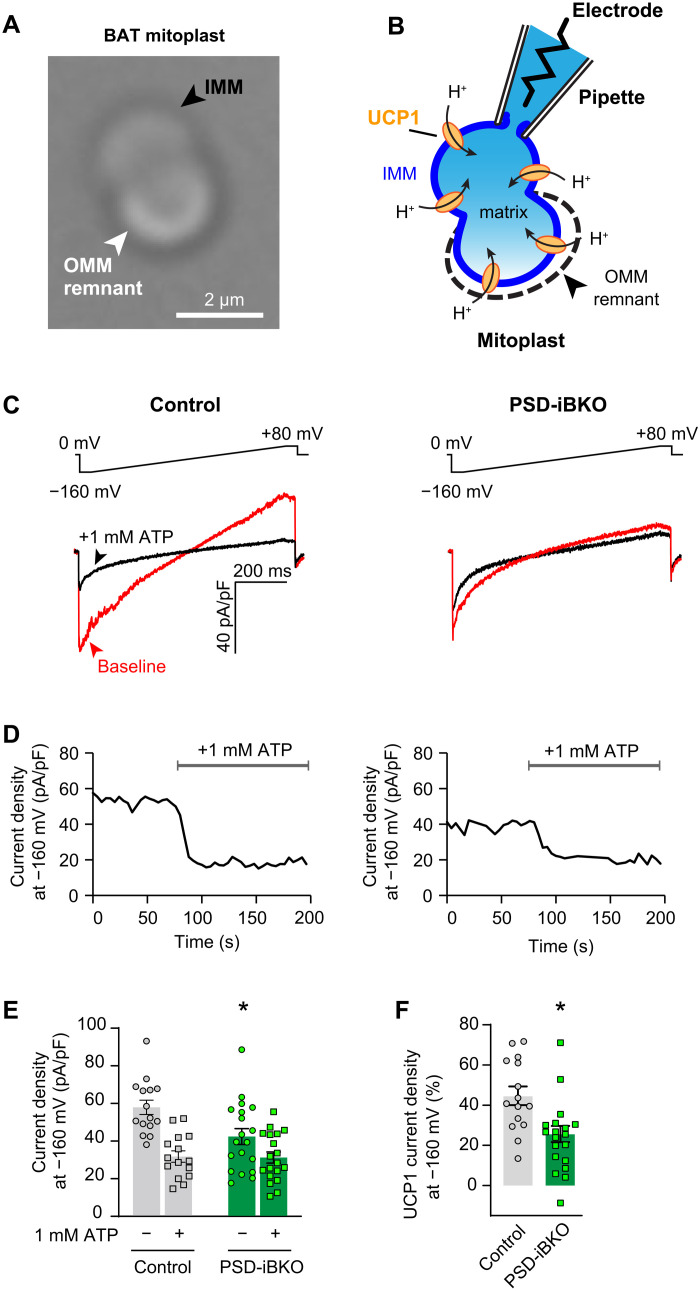
Mitochondrial PE is essential for proton current through UCP1. (**A**) Differential interference contrast image of BAT mitoplasts reveals typical bilobed appearance. IMM, black arrowhead; OMM remnant, white arrowhead. Scale bar, 2 μM. (**B**) Schematic illustrating electrophysiological recording setup for proton (H^+^) current. (**C**) Top: Voltage ramp protocol. Bottom: Exemplar traces showing baseline proton currents (red) and after the addition of 1 mM ATP (black). Measurements are taken at −160 mV (arrowheads). (**D**) Exemplar time course of proton current inhibition with ATP. (**E**) Summary of proton current densities (*n* = 15 to 19 mitoplasts per group). (**F**) Quantification of UCP1 current density, taken as the difference for each mitoplast between baseline and after ATP addition divided by total current density, from (E) (*n* = 15 to 19 mitoplasts per group). Data are presented as ±SEM.

To more directly assess the effect of PE on UCP1 activity, we quantified UCP1-dependent proton current in mitoplasts prepared from BAT mitochondria in control and PSD-iBKO mice (Fig. 6A) (*45*, *46*). IMM portion of mitoplasts were patch-clamped to perform electrophysiologic measurements of proton current through UCP1 (Fig. 6B). The UCP1 contribution to the proton current density is the portion of the total baseline current that is inhibited by the subsequent addition of 1 mM ATP (Fig. 6, C and D). Notably, total proton current was substantially and consistently diminished in BAT mitoplasts from PSD-iBKO mice compared to control mice, whereas the difference was abolished after addition of ATP (Fig. 6E). Quantifying UCP1 proton current density as the percentage attributable to UCP1 revealed significantly lower levels in mitoplasts from PSD-iBKO mice compared to control mice (Fig. 6F). Thus, while UCP1 levels within mitochondria were unchanged, its activity was clearly reduced, suggesting that mitochondrial PE can facilitate UCP1 activity.

## DISCUSSION

OXPHOS is the process whereby energy derived from substrates is transduced by a series of reactions that occur in and across the IMM to ultimately yield ATP synthesis. UCP1, the chief enzyme of brown adipose thermogenesis, also resides in IMM where it disrupts the proton gradient to uncouple ATP synthesis from the ETS. Here, we provide evidence that mitochondrial PE is an energy-responsive IMM metabolite that alters UCP1 activity to regulate thermogenesis. At the organism level, mice with reduced mitochondrial PE were cold intolerant and insensitive to β3 agonist–induced increase in whole-body oxygen consumption. Deep phenotyping of BAT mitochondria revealed that low PE robustly reduces UCP1-dependent respiration and UCP1 proton current. Together, these findings suggest that mitochondrial PE responds to changes in BAT thermogenic demand to potentiate UCP1 activity.

Previously, we demonstrated that adipose CL is essential for BAT thermogenesis and systemic energy homeostasis (*15*). CL has been implicated in mitochondrial function in various tissues including the liver, skeletal muscle, and BAT (*20*, *47*–*49*). As a lipid that is almost exclusively localized in IMM, it would be reasonable to suspect that CL might directly affect UCP1 function to uncouple OXPHOS. Some studies suggest that CL stabilizes UCP1 (*50*), while other studies suggest that CL attenuates inhibition of UCP1 by nucleotide binding (*51*). However, we found that although CLS deletion indeed made mice cold intolerant, it had no effect on UCP1-dependent respiration. Nonetheless, it is important to acknowledge that our strategy with CLS knockout did not achieve a complete deprivation of CL in the IMM milieu. Mitochondrial CL levels also did not decrease with thermoneutrality or UCP1 knockout, suggesting that CL levels do not universally respond to changes in BAT thermogenic burden. Other studies suggest that CL acutely responds to changes in diet, and cold exposure is known to increase food intake (*52*, *53*).

What is the mechanism by which CL regulates thermogenesis independent of UCP1? In our studies, we ruled out an effect of CL on BAT mitochondrial density, cristae morphology, or UCP1 protein abundance. We also ruled out the possibility that CL alters the sensitivity of UCP1 to be activated by fatty acids or changes in GPCR signaling that induces fatty acid mobilization. Our previous study suggests that CLS has a robust effect on modulating transcription (*15*), and we continue to subscribe to this idea that CL or CLS may have an alternate role to regulate metabolism independent of mitochondria. Nevertheless, CLS deletion did not reduce BAT thermogenesis by altering protein abundance of enzymes involved in calcium, creatine, or lipid cycling. It remains possible that CLS has a more nuanced influence on these pathways or acts on other uncharacterized mechanisms.

Unlike CL, mitochondrial PE responded to cold, thermoneutrality, and UCP1 knockout, making this lipid a candidate for an energy-responsive UCP1 rheostat. Decreased mitochondrial PE directly reduced UCP1 proton current, without altering electron leak or ATP synthesis. The reduction in UCP1 activity occurred in the absence of changes in UCP1 protein content per unit of mitochondria. This suggests that PE may activate UCP1 function. How does mitochondrial PE modulate UCP1? Unlike CL, PE has not been implicated to bind to UCP1 (*50*). However, a study with UCP1-reconstituted liposomes suggests that PE enhances the UCP1 proton translocation (*54*). The proposed mechanism was that PE adducts alter the membrane boundary potential of the IMM bilayer to facilitate the protonophoric activity of UCP1. We note that reduced levels of PE coincided with robustly deformed cristae, an observation consistent with their model on the effect that PE has on membrane curvature. Regardless of the mechanism, compromised UCP1 function with low mitochondrial PE was sufficient to impair thermogenesis induced by cold or β3 agonist administration. Together with our data that cold or thermoneutrality regulates the abundance of PE in mitochondria, we postulate that PE-dependent modulation of UCP1 function is a physiologically relevant mechanism for thermogenesis.

We also attempted to increase mitochondrial PE content to see whether it would be sufficient to increase UCP1 function. In summary, both our in vivo and in vitro approaches failed to sufficiently increase mitochondrial PE in brown adipocytes. For in vivo experiments, we performed PSD overexpression by examining mice with conditional PSD overexpression using UCP1-Cre (PSD-BKI; fig. S7, A to M). We previously used this strategy to successfully increase PSD expression and mitochondrial PE in the skeletal muscle (*4*). While PSD transcript was successfully elevated in BAT from PSD-BKI mice compared to control mice, this intervention only increased mitochondrial PE by ~10%, likely due to endogenous PSD activity that is already high in BAT. Further, we did not observe any differences in β3 agonist–induced oxygen consumption or UCP1-dependent respiration. Similarly, in vitro ethanolamine supplementation was not effective in increasing mitochondrial PE content, and doses of lyso-PE supplementation that were sufficient to increase mitochondrial PE also induced cell death. Last, we performed experiments using small unilamellar vesicles (SUVs) in an attempt to directly deliver PE to mitochondria. However, PE alone does not effectively form SUVs, and fusion of PE/PC SUVs at various ratios all substantially diluted mitochondrial proteins and lowered total mitochondrial respiration. Thus, at this point in time, we are unable to provide evidence for gain of function of PSD or mitochondrial PE to increase UCP1 function.

How do changes in temperature regulate PE and other mitochondrial lipids? Our data suggest that mitochondrial PE is at least partly regulated by changes in PSD transcription. There is very little known regarding the transcriptional control of enzymes for mitochondrial lipid biosynthesis and transport. In the skeletal muscle, we previously showed that exercise or sedentariness influences PSD to regulate mitochondrial PE level (*4*). Thus, we suspect that the PSD-mitochondrial PE axis bidirectionally responds to changes in energy demand to optimize mitochondrial bioenergetics in multiple cell types. Conversely, we suspect that the CLS-mitochondrial CL axis responds to changes in energy supply. Dietary interventions such as high-fat diet feeding and calorie restriction are known to influence cellular CL levels in multiple tissues (*55*–*59*). We have preliminary evidence in nonadipose tissues that mitochondria CL specifically responds to diet interventions. In the current study, cold intervention increased mitochondrial CL content, which is known to also increase food intake in mice (*52*, *53*, *60*). These lines of evidence provide important insights into a more global understanding of how mitochondrial lipids such as PE and CL respond to energy demand or supply to influence mitochondrial energetics.

UCP1-dependent thermogenesis remains an important futile energy process to understand in the context of metabolic health. In this study, we identified an important role that mitochondrial PE plays in UCP1-dependent thermogenesis in BAT. CL did not appear to directly regulate UCP1 function, although it is clear that the CLS-CL axis plays a role in regulating thermogenesis that is likely independent of mitochondrial UCP1 activity. Combined with our previous findings, we propose that mitochondrial PE is a universal cellular rheostat that modulates mitochondrial efficiency in response to changes in energy demand.

## MATERIALS AND METHODS

### Experimental design

To examine how BAT mitochondrial lipidome might be influenced by changes in thermogenic burden, we studied mice housed in cold, thermoneutrality, or with UCP1 deletion. We also used high-resolution respirometry and fluorometry to study OXPHOS and UCP1 bioenergetics in BAT mitochondria. To study the role of mitochondrial CL or PE in BAT, mice with BAT-specific deletion of CLS or PSD were generated. These mice were then subjected to whole-body and BAT mitochondrial phenotyping. Whole-body phenotyping included cold tolerance testing and measurements of whole-body oxygen consumption with or without CL-316,243. Mitochondrial phenotyping included electron microscopy, histological staining, Western blotting, and high-resolution respirometry and fluorometry. Lastly, UCP1 proton current was measured in PE-deficient mitoplasts via patch clamp with and without ATP inhibition.

### Rodent models

The animal studies performed here were approved by the University of Utah Institutional Animal Care and Use Committee. All mice used in this study were of the C57Bl/6J background. Unless otherwise noted, experiments were performed on mice 12 weeks of age that were housed at an ambient temperature of 22°C. They were fed a standard chow diet (Teklad, 2920X). Transgenic animals were injected with 15 mg/kg of tamoxifen (Sigma-Aldrich, T5648) dissolved in sunflower oil (Sigma-Aldrich, S5007) for 5 days in a row 3 weeks (PSD-iBKO) or 4 weeks (CLS-iBKO) before sacrifice unless otherwise noted. Males and females were used for each experiment, and no sex-dependent differences were observed. The mice were fasted for 4 hours before sacrifice using ketamine/xylazine (MWI Animal Health, 501090).

#### Wild-type mice

C57BL/6J mice stock no. 000664 were obtained from The Jackson Laboratory.

#### CLS-iBKO

This study used tamoxifen-inducible BAT-specific CLS knockout (CLS-iBKO) mice generated by E.G.S. and Z.G.-H. (University of Copenhagen) (*15*). This line was later mated to a UCP1-specific Cre-ERT2 driver (UCP1 Cre-ERT2^+/−^) generated by C. Wolfrum (ETH Zürich) (*61*). The resulting (CLS-cKO^+/+^, UCP1 Cre-ERT2^+/−^; designated as CLS-iBKO) and littermate control (CLS-cKO^+/+^, no Cre) mice were used in this study. Both groups were administered tamoxifen.

#### PSD-iBKO

Tamoxifen-inducible PSD knockout in BAT (PSD-iBKO) mice were generated for this study. The PSD conditional knockout mice were previously generated by the Funai laboratory (*4*). This line was then mated to the same UCP1-specific Cre-ERT2 driver (UCP1 Cre-ERT2^+/−^) from C. Wolfrum’s laboratory. Tamoxifen-injected PSD-iBKO (PSD-cKO^+/+^, UCP1 CreERT2^+/−^; designated as PSD-iBKO) and littermate control (PSD-cKO^+/+^, no Cre) mice were used in this study.

#### PSD-BKI

The BAT PSD knock-in (PSD-cKI^+/+^) mouse line was generated by inserting Myc-tagged *Pisd* cDNA into a Rosa26 locus. The gene is preceded by a CAG promotor and a stop codon flanked by loxP sites to allow for tissue specific expression (lox-STOP-lox). The PSD-BKI line was created by mating PSD-cKI^+/+^ mice to mice with a constitutive UCP1-Cre driver (UCP1Cre^+/−^) obtained from The Jackson Laboratory, stock no. 024670 (*62*). This cross generated PSD-BKI (PSD-cKI^+/−^, UCP1Cre^+/−^; designated PSD-BKI) and littermate control (PSD-cKI^+/−^, no Cre) mice.

### Mitochondrial isolation

Mitochondrial isolation from BAT harvested from mice that were euthanized 4 hours after fasting was performed as previously described (*63*). BAT from freshly sacrificed mice was finely minced in pH 7.4 mitochondrial isolation media (MIM; 300 mM sucrose, 10 mM Hepes, and 1 mM EGTA) with bovine serum albumin (BSA; 1 mg/ml; Sigma-Aldrich, S7903, H3375, E3889, and A7030, respectively). The mixture was homogenized at 1000 rpm for six to eight passes using an overhead stirrer (Thermo Fisher Scientific, 14-500-210). Homogenates were centrifuged at 10,000*g* for 10 min. The supernatant was discarded, and the samples were centrifuged at 200*g* for 5 min twice. Each time, the supernatant was collected (the pellet discarded) and transferred to a new tube. The supernatant was centrifuged once more at 10,000*g* for 10 min. The supernatant was discarded, and the pellet was resuspended in MIM. Protein concentration was assessed using the Pierce BCA protein assay kit (Thermo Fisher Scientific, 23227).

### Respirometry

Mitochondrial oxygen consumption was measured using Oroboros oxygraphs (Oroboros Instruments, 10023-01). Resorufin or the reduced form of nicotinamide adenine dinucleotide phosphate (NADPH) production was measured using FluoroMax-4 (Horiba Scientific) for H_2_O_2_ and ATP assays, respectively. Experiments were performed in buffer Z [105 mM K-MES (Sigma-Aldrich, M0895), 30 mM KCl, 1 mM EGTA, 10 mM K_2_HPO_4_ (Sigma-Aldrich, P5655), 5 mM MgCl_2_-6H_2_O, and BSA (5 mg/ml) (pH 7.4)] as previously described (*64*). UCP1-dependent respiration was stimulated by treating mitochondria with 0.5 mM malate (Sigma-Aldrich, M7397) and 5 mM pyruvate (Sigma-Aldrich, P2256) or carnitine (Sigma-Aldrich, 8.40092) and palmitoyl-CoA (Sigma-Aldrich P9716) or palmitoyl-l-carnitine (P1645). UCP1 was inhibited using 4 mM guanosine diphosphate (GDP) (Sigma-Aldrich, G7127). ATP production was indirectly measured using an enzymatically coupled reaction that produced NADPH. NADPH was excited at 340 nm, and fluorescence was measured at 460 nm every 2 s as previously described (*65*). ATP synthesis was stimulated by treating mitochondria with 0.5 mM malate, 5 mM pyruvate, 5 mM glutamate (Sigma-Aldrich, G5889), and 5 mM succinate (Sigma-Aldrich, S3674) with 2, 20, and 200 μM ADP (Sigma-Aldrich, A5285). ADP-stimulated respiration includes all components of respiration including UCP1-dependent and ATP-linked respiration. In BAT mitochondria, ADP not only stimulates ATP-linked respiration but also partially inhibits UCP1. Maximal ADP inhibition of UCP1 occurs at low mM concentrations, so the effect of ADP to suppress UCP1 is relatively little at 2 and 20 μM concentrations, while the effect is more impactful at the 200 μM concentration. The ATP/O ratio was calculated by coupling the FluoroMax ATP assay with an identical assay performed in the Oroboros oxygraphs. H_2_O_2_ production in isolated mitochondria was stimulated using 10 mM succinate, followed by 1 μM auranofin (Sigma-Aldrich, A6733) and 100 μM carmustine (BCNU) (Sigma-Aldrich, C0400). Resorufin is produced at a 1:1 ratio with H_2_O_2_ in the presence of Amplex Red (Thermo Fisher Scientific, A12222), superoxide dismutase (Sigma-Aldrich, S7446), and horseradish peroxidase (Sigma-Aldrich, P8375). It was excited at 550 nm, and fluorescence was measured at 585 nm.

### Lipidomics

Mitochondrial phospholipids were extracted from isolated mitochondria using a modified Matyash lipid extraction protocol (*66*). Phospholipid internal standards (SPLASH Mix Avanti Polar Lipids 330707 and Cardiolipin Mix I Avanti Polar Lipids LM6003) and 50 μg of protein from isolated mitochondria were added to ice-cold 3:10 methanol:methyl-*tert*-butyl-ether. Samples were vortexed and sonicated for 1 min before being incubated on ice for 15 min. During this time, samples were vortexed every 5 min. H_2_O was added, and the samples were again incubated on ice for 15 min with vortexing taking place every 5 min. The samples were centrifuged at 15,000*g* for 10 min. The supernatant was collected, and the solvent evaporated using a SpeedVac set to 37°C for 1 hour. The lipid pellet was resuspended using a 9:1 methanol:toluene mixture. Phospholipid analysis was conducted using liquid chromatography–mass spectrometry on an Agilent 6530 ultraperformance liquid chromatography–quadrupole time-of-flight mass spectrometer.

### Metabolic phenotyping

Cold tolerance testing was conducted on single housed mice at 4°C with access to water and light bedding. Core body temperature was measured using a temperature-sensitive transponder injected into dorsal subcutaneous adipose tissue (Bio Medic Data Systems, IPTT 300). The transponder was placed in the mice 1 week before cold tolerance testing. Transponder readings were assessed using a Reader-Programmer (Bio Medic Data Systems, DAS 8007). Whole-body VO_2_ was measured using the Comprehensive Lab Animal Monitoring System (Columbus Instruments). Intraperitoneal injected CL 316,243 (Sigma-Aldrich, C5976) was used to stimulate BAT activity.

### Electron microscopy

BAT was isolated from mice, minced with scissors, and fixed in ice-cold 2.5% glutaraldehyde. After incubating for 48 hours at 4°C, the tissue was delivered to the University of Utah Electron Microscopy Core for after fixation, dehydration, and embedding. The embedded tissues were sectioned and stained using uranyl acetate. The tissues were then imaged with a JEOL JEM1400 Plus transmission electron microscope and acquired using a Soft Imaging Systems MegaView III charge-coupled device camera.

### Gene expression

BAT stored at −80°C was thawed and placed in 1 ml of TRIzol (Thermo Fisher Scientific, 15596018). The tissue was homogenized and spun down at 10,000*g* for 10 min. The lipid layer was discarded, and 200 μl of chloroform was added to the tube. The tube was mixed by inverting it 10 times. After 2 min, samples were centrifuged at 15,000*g* for 15 min at 4°C. The aqueous supernatant was collected and placed in a new tube containing 500 μl of 100% isopropyl alcohol. The mixture was inverted a few times and incubated at RT for 10 min. The samples were spun down at 15,000*g* for 10 min at 4°C. The supernatant was carefully aspirated such that the pellet was not disturbed. The pellet was washed with 75% ethyl alcohol and spun down at 5500*g* for 5 min. The supernatant was aspirated, and the pellet was air dried for 10 min before being resuspended in tris-EDTA buffer. A cDNA library was generated by reverse-transcribing the RNA using the iScript cDNA Synthesis Kit (Bio-Rad, 1708891).

In contrast to gene expression, relative mtDNA abundance was measured using genomic DNA. Genomic DNA was obtained by processing frozen BAT with the Qiagen DNeasy Blood and Tissue Kit (Qiagen, 69504).

For quantitative polymerase chain reaction, equal nanograms of cDNA or genomic DNA were combined with SYBR Green (Thermo Fisher Scientific, A25776) and gene-specific primers and then placed in a 384-well plate. Primer sequences were obtained from the Primer Bank (https://pga.mgh.harvard.edu/primerbank/). Gene expression was analyzed using a QuantStudio 12K Flex (Life Technologies). Unless otherwise indicated, mRNA levels were normalized to RPL32.

### Histological analysis

BAT was fixed by placing the tissue in a 4% paraformaldehyde–phosphate-buffered saline solution for 48 hours. The tissue was then placed in 70% ethanol for another 48 hours. The tissue was embedded in paraffin, cut into approximately 5-μm pieces, and stained using hematoxylin and eosin or Masson’s trichrome blue. The sectioned tissues were then imaged using an Axio Scan.Z1 (Zeiss).

### Protein analysis

For whole-cell analysis, BAT stored at −80°C was thawed and placed in ice-cold homogenization buffer (150 mM NaCl, 50 mM tris-HCl, 5 mM EDTA, 1% Triton X-100, 0.1% SDS, 0.1% sodium deoxycholate, and 1% protease and phosphatase inhibitor cocktail were added immediately before use) (Thermo Fisher Scientific, 78446). The sample was homogenized and centrifuged at 12,000*g* for 5 min. The concentration of protein was determined using a BCA (details included above).

For analyzing proteins in isolated mitochondria, mitochondria were first isolated from BAT using the protocol outlined above. From this point forward, identical protocols were followed. Equal quantities of protein were mixed with Laemmli sample buffer and loaded into a gradient SDS–polyacrylamide gel electrophoresis gel (Bio-Rad, 4561086). The proteins were transferred from the gel onto nitrocellulose membranes (Bio-Rad, 1620097). Membranes were blocked using 5% BSA in tris-buffered saline with 0.1% Tween 20 (TBST) for 1 hour before being treated with primary antibodies overnight at 4°C. The following primary antibodies were used: UCP1 (Alpha Diagnostics, UCP11-A), total OXPHOS antibody cocktail (Abcam, MS604-300), citrate synthase (Abcam, Ab96600), PISD (Sigma-Aldrich, HPA031091), HSL (Cell Signaling Technology, 4107S), HSL Ser^563^ (Cell Signaling Technology, 4139), HSL Ser^660^ (Cell Signaling Technology, 4126), ATGL (Cell Signaling Technology, 2439S), ACC (Cell Signaling Technology, 3662), FAS (3180), DGAT1 (Thermo Fisher Scientific, PA5-117074), DGAT2 (Abcam, 237613), RyR2 (Sigma-Aldrich, AB9080), SERCA2 (Abcam, 3625), TNAP (Abcam, 126820), CKB (Abcam, 92452), CKMT2 (Thermo Fisher Scientific, 13207-1-AP), AAC1 (Abcam, 220408), and AAC2 (Cell Signaling Technology, 14671). The membranes were washed five times using TBST and placed in secondary antibodies at a concentration of 1:5000 in 5% nonfat dry milk (NFDM) or BSA for 1 hour. The membranes were washed five times in TBST and twice in TBS before imaging. Immediately before imaging, Western Lightning Plus, Chemiluminescent substrate (PerkinElmer, NEL 104001EA) was pipetted onto the membrane. Membranes were imaged using a FluorChem E imager (ProteinSimple).

### Isolation of BAT mitoplasts

Five-week-old mice were sacrificed (CO_2_ asphyxiation) followed by cervical dislocation. Interscapular BAT was manually separated, and mitochondria were isolated as described in (*45*). Tissue was disrupted with a Potter-Elvehjem homogenizer, and a crude mitochondrial fraction was isolated by differential centrifugation. Mitoplasts were generated from BAT mitochondria using a French press set at 1200 psi to mechanically break the OMM. For recording, mitoplasts were aliquoted into a divalent-free KCl (DVF KCl) solution containing 150 mM KCl, 10 mM Hepes, and 1 mM EGTA (pH 7.2 with KOH) and plated on 5-mm coverslips coated with 0.1% gelatin.

### Whole-mitoplast electrophysiology

Mitoplasts (3 to 5 μm) had typical membrane capacitances of 0.5 to 1.0 pF. Five- to 10-gigaohm seals were formed in DVF KCl, where a fast voltage step of 250 to 600 mV was applied to break in. Entering the whole-mitoplast configuration was monitored by changes in the amplitude of the capacitance transients. Mitoplasts were interrogated every 4 s with a ramp protocol from −160 to +80 mV, at a holding potential of 0 mV. Recording borosilicate pipettes (15- to 25-megohm resistance) were filled with 150 mM tetraethylammonium hydroxide, 1.5 mM EGTA, 1.0 mM magnesium gluconate, 150 mM Hepes, and 2 mM tris-Cl (pH adjusted to 7.0 with d-gluconic acid, tonicity adjusted to 325 to 350 mmol/kg with sucrose). After establishing whole-mitoplast configuration in KCl DVF solution, the bath solution was changed to a proton current recording solution containing 150 mM Hepes, 1 mM EGTA, and 0.5 mM MgCl_2_ (pH adjusted to 7.0 with tris-base, tonicity adjusted to 300 mmol/kg with sucrose). ATP (Sigma-Aldrich, 9187) was used to inhibit UCP1. Data acquisition and analysis were performed using PClamp 10 (Molecular Devices) and Origin 7.5 (Origin Lab). Electrophysiological data were acquired at 10 kHz and filtered at 0.5 to 1 kHz. Images were prepared on Adobe Illustrator. For presentation purposes of exemplars, these have been subjected to the Simplify filter to reduce file size without changing shape, and fast capacitance transients have been removed.

### Statistical analysis

Data and power analyses were performed using GraphPad Prism 9.3 software. All experiments were randomized and blinded where appropriate. The value of *n* for each experiment is noted in the figure legends and corresponds to the data obtained from an individual mouse or batch of cells. All data are presented as means ± SEM. Significance was set at *P* < 0.05. Data with only two groups were analyzed using two-tailed Student’s *t* test. Data with more than two groups were analyzed using two-way analysis of variance (ANOVA).

## Notes

View/request a protocol for this paper from *Bio-protocol*.
