# Supplementary material for: Mitochondrial phosphatidylethanolamine modulates UCP1 to promote brown adipose thermogenesis
Source: Sci Adv. 2023 Feb 24;9(8):eade7864. doi: 10.1126/sciadv.ade7864 (PMC9956115; doi:10.1126/sciadv.ade7864)
Supplement: Supplementary file 1 — Figs. S1 to S7 [file sciadv.ade7864_sm.pdf]

Science Advances
AAAS

Supplementary Materials for

Mitochondrial phosphatidylethanolamine modulates UCP1 to promote brown adipose thermogenesis

Jordan M. Johnson *et al.*

Corresponding author: Katsuhiko Funai, kfunai@utah.edu

Sci. Adv. **9**, eade7864 (2023)
DOI: 10.1126/sciadv.ade7864

This PDF file includes:

Figs. S1 to S7

Supplemental Figure 1

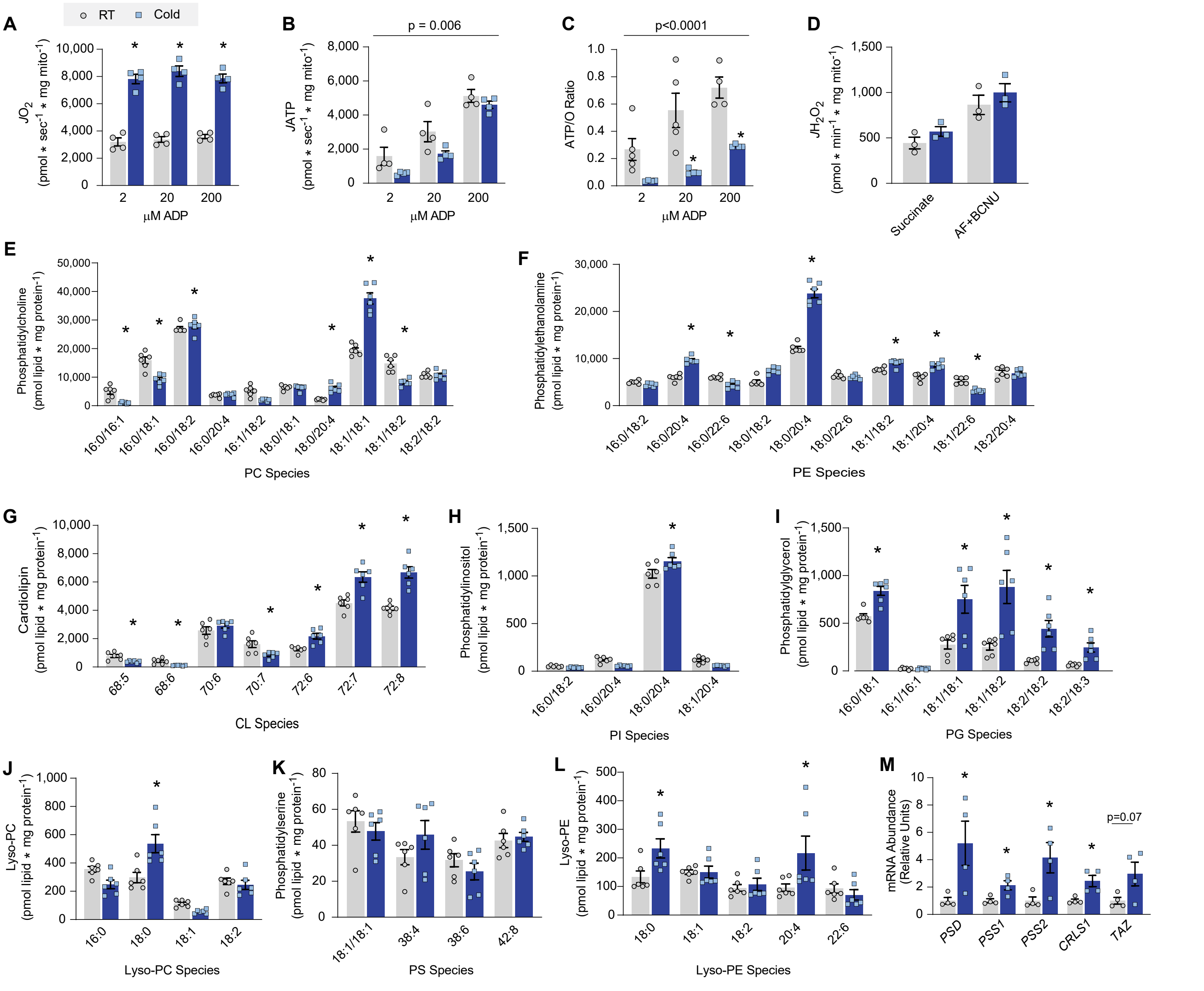

Figure S1. Cold-induced adaptations in mitochondrial bioenergetics and phospholipids in BAT. (**A**) Mitochondrial O_2 consumption in BAT from C57BL/6J mice housed at RT or 6.5 °C for 7 days, measured in the presence of 5 mM pyruvate, 0.2 mM malate, 5 mM glutamate, 5 mM succinate, and 2, 20, and 200 µM ADP from. n=4/group. (**B**) ATP production in the presence of 5 mM pyruvate, 0.2 mM malate, 5 mM glutamate, 5 mM succinate, and 2, 20, and 200 µM ADP. n=4/group. (**C**) Mitochondrial coupling efficiency (ATP/O ratio) of BAT mitochondria in C57BL/6J mice housed at RT or 6.5 °C for 7 days. n=4/group. (**D**) H_2O_2 production in BAT mitochondria from C57BL/6J mice housed at RT and 6.5 °C, measured in the presence of 10 mM succinate and antioxidant inhibitors auranofin (AF) and carmustine (BCNU). n=4/group. (**E-L**) Mass spectrometric analyses of mitochondrial lipids in BAT from C57BL/6J mice housed at RT and 6.5 °C. PC (**E**), PE (**F**), CL (**G**), PI (**H**), PG (**I**), lyso-PC (**J**), PS (**K**), and lyso-PE (**L**). n=6/group. (**M**) mRNA abundance of genes of mitochondrial PE and CL biosynthesis. n=4/group. Data are presented as ± S.E.M. $*p < 0.05$.

Supplemental Figure 2

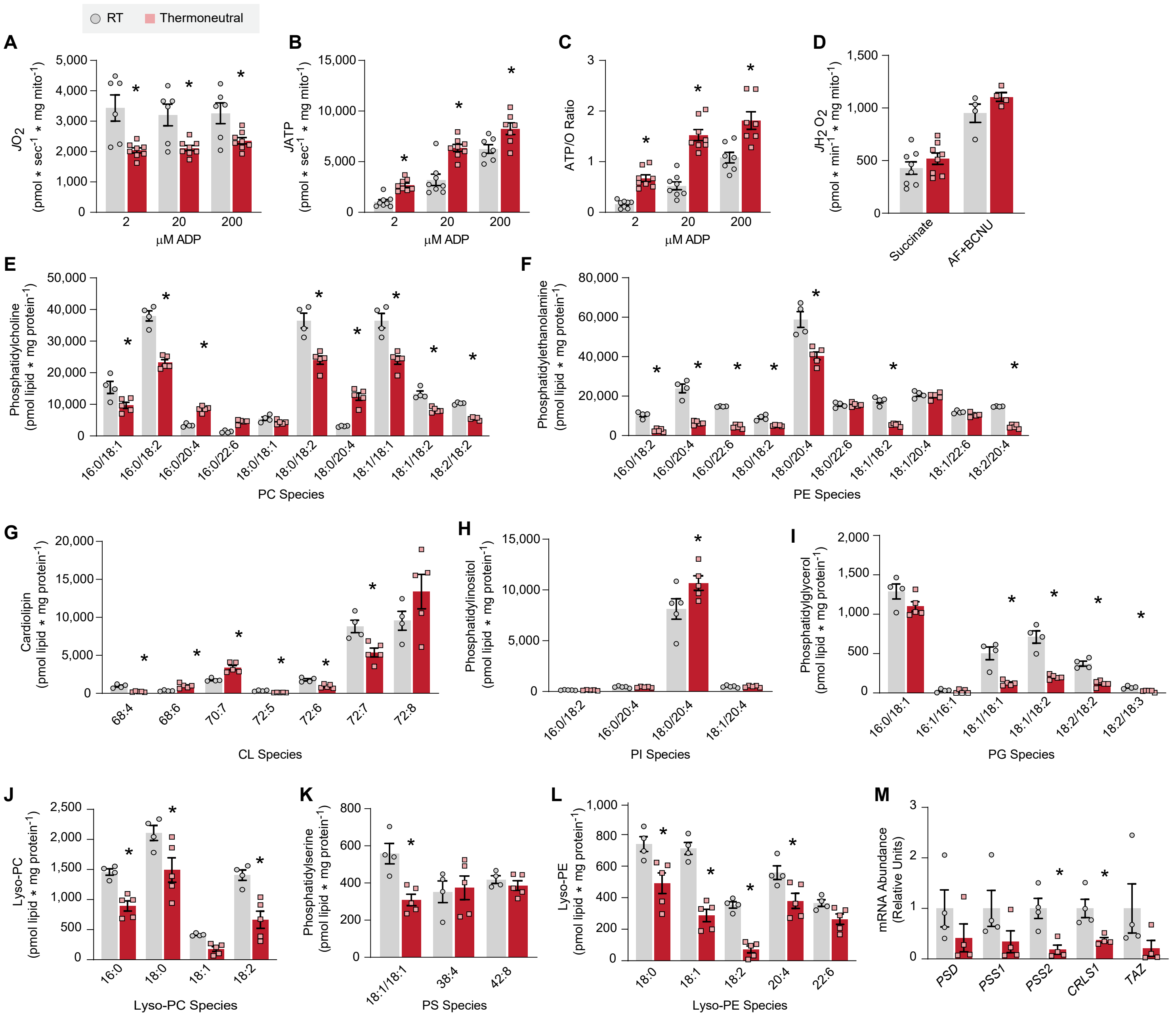

Figure S2. Thermoneutrality-induced adaptations in mitochondrial bioenergetics and phospholipids in BAT. (**A**) Mitochondrial O_2 consumption in BAT from C57BL/6J mice housed at RT or 30 °C for 30 days, measured in the presence of 5 mM pyruvate, 0.2 mM malate, 5 mM glutamate, 5 mM succinate, and 2, 20, and 200 μM ADP from. n=6-8/group. (**B**) ATP production in the presence of 5 mM pyruvate, 0.2 mM malate, 5 mM glutamate, 5 mM succinate, and 2, 20, and 200 μM ADP. n=8/group. (**C**) Mitochondrial coupling efficiency (ATP/O ratio) of BAT mitochondria. n=8/group. (**D**) H_2O_2 production in the presence of 10 mM succinate and antioxidant inhibitors auranofin (AF) and carmustine (BCNU). n=4-8/group. (**E**-**L**) Mass spectrometric analyses of mitochondrial lipids in BAT from C57BL/6J mice housed at RT and 30 °C. PC (**E**), PE (**F**), CL (**G**), PI (**H**), PG (**I**), lyso-PC (**J**), PS (**K**), and lyso-PE (**L**). n=4-5/group. (**M**) mRNA abundance of genes of mitochondrial PE and CL biosynthesis. n=4/group. Data are presented as ± S.E.M. *$p < 0.05$.

Supplemental Figure 3

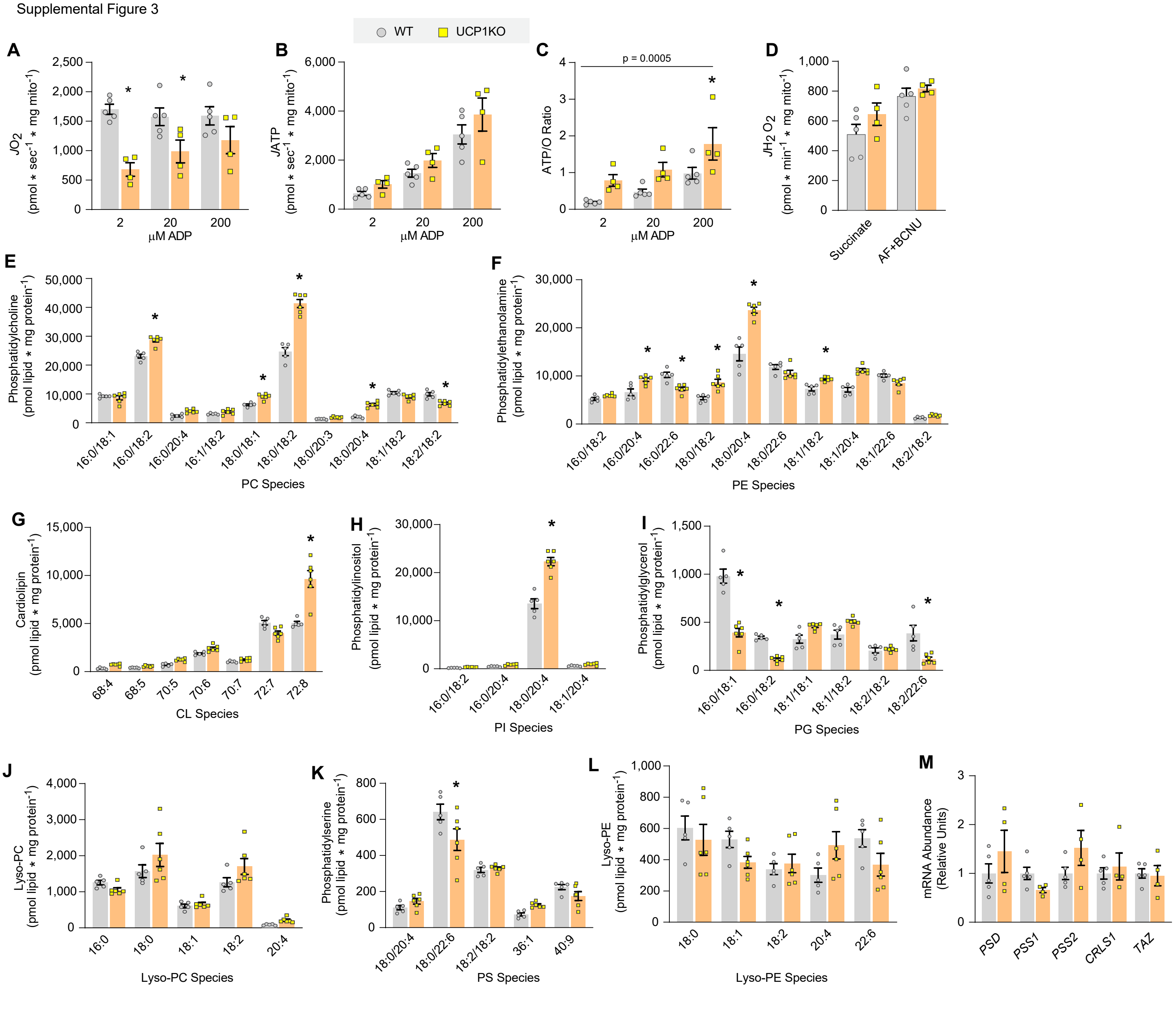

Figure S3. Effects of UCP1 deletion on mitochondrial bioenergetics and phospholipids in BAT. (**A**) Mitochondrial O_2 consumption in BAT from WT and UCP1KO mice, measured in the presence of 5 mM pyruvate, 0.2 mM malate, 5 mM glutamate, 5 mM succinate, and 2, 20, and 200 µM ADP. n=4-5/group. (**B**) ATP production in the presence of 5 mM pyruvate, 0.2 mM malate, 5 mM glutamate, 5 mM succinate, and 2, 20, and 200 µM ADP. n=4-5/group. (**C**) Mitochondrial coupling efficiency (ATP/O ratio) of BAT mitochondria. n=4-5/group. (**D**) H_2O_2 production in the presence of 10 mM succinate and antioxidant inhibitors auranofin (AF) and carmustine (BCNU). n=4-5/group. (**E**-**L**) Mass spectrometric analyses of mitochondrial lipids in BAT from WT and UCP1KO mice. PC (**E**), PE (**F**), CL (**G**), PI (**H**), PG (**I**), lyso-PC (**J**), PS (**K**), and lyso-PE (**L**). n=5-6/group. (**M**) mRNA abundance of genes of mitochondrial PE and CL biosynthesis. n=4-5/group. Data are presented as ± S.E.M. *$p < 0.05$.

Supplemental Figure 4

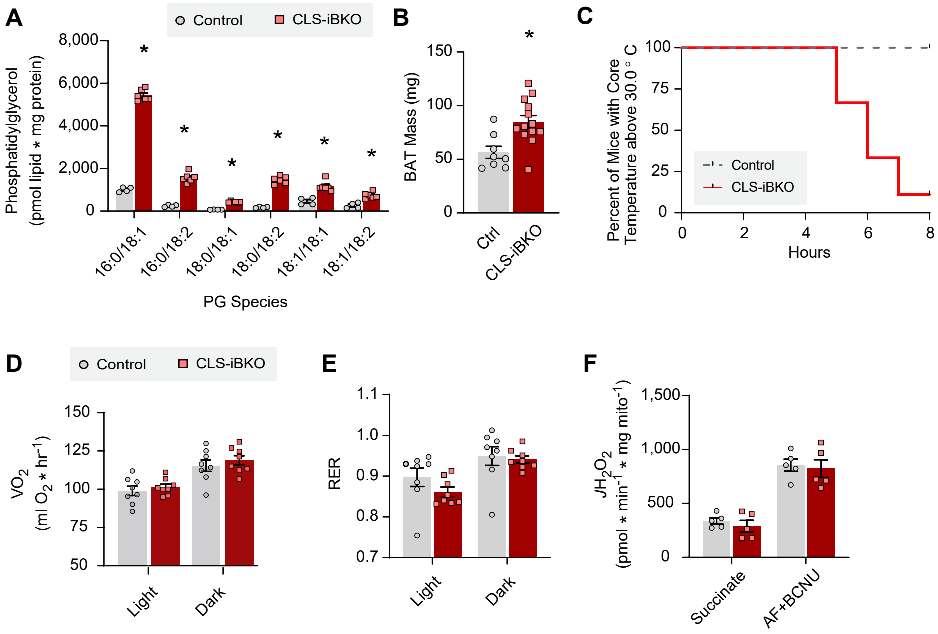

Figure S4. Additional data on CLS-iBKO mice. (**A**) Mitochondrial PG, a precursor to mitochondrial CL. n=4-6/group. (**B**) BAT mass. n=8-13/group. (**C**) Percent of mice with core body temperature above 30.0 °C during cold tolerance testing. n=7-9/group. (**D**) Whole-body oxygen consumption in metabolic cage. n=8/group. (**E**) Respiratory exchange ratio (RER) in metabolic cage. n=8/group. (**F**) Mitochondrial H_2O_2 production in the presence of 10 mM succinate and antioxidant inhibitors auranofin (AF) and carmustine (BCNU). n=5/group. Data are presented as ± S.E.M. *$p < 0.05$.

Supplemental Figure 5

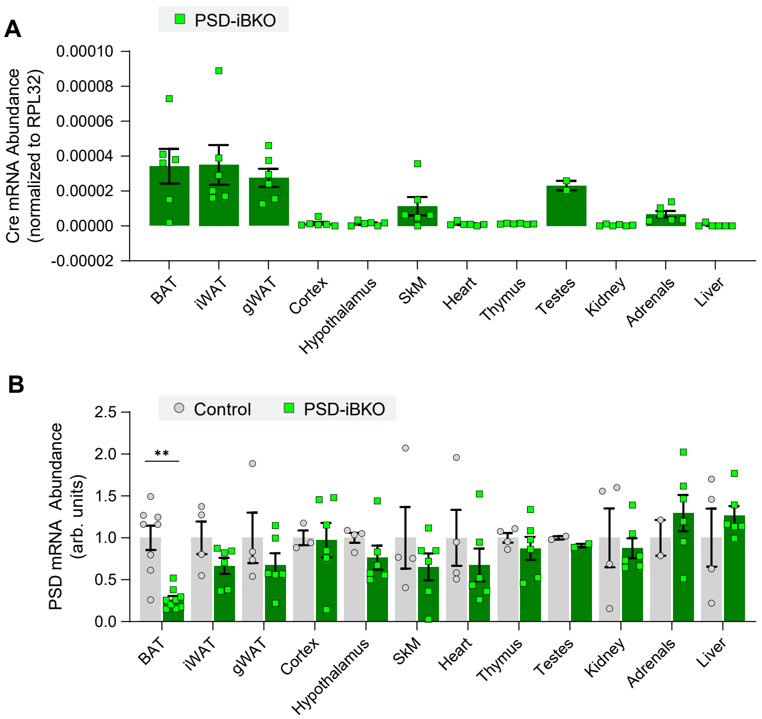

Figure S5. UCP1-CreERT2 expression does not result in decreased PSD expression outside of thermogenic adipose tissue. (**A**) Cre mRNA abundance in various tissues of PSD-iBKO mice. n=2-6/group. (**B**) PSD mRNA abundance in various tissues of control and PSD-iBKO mice. n=2-10/group. Data are presented as ± S.E.M. *$p < 0.05$.

Supplemental Figure 6

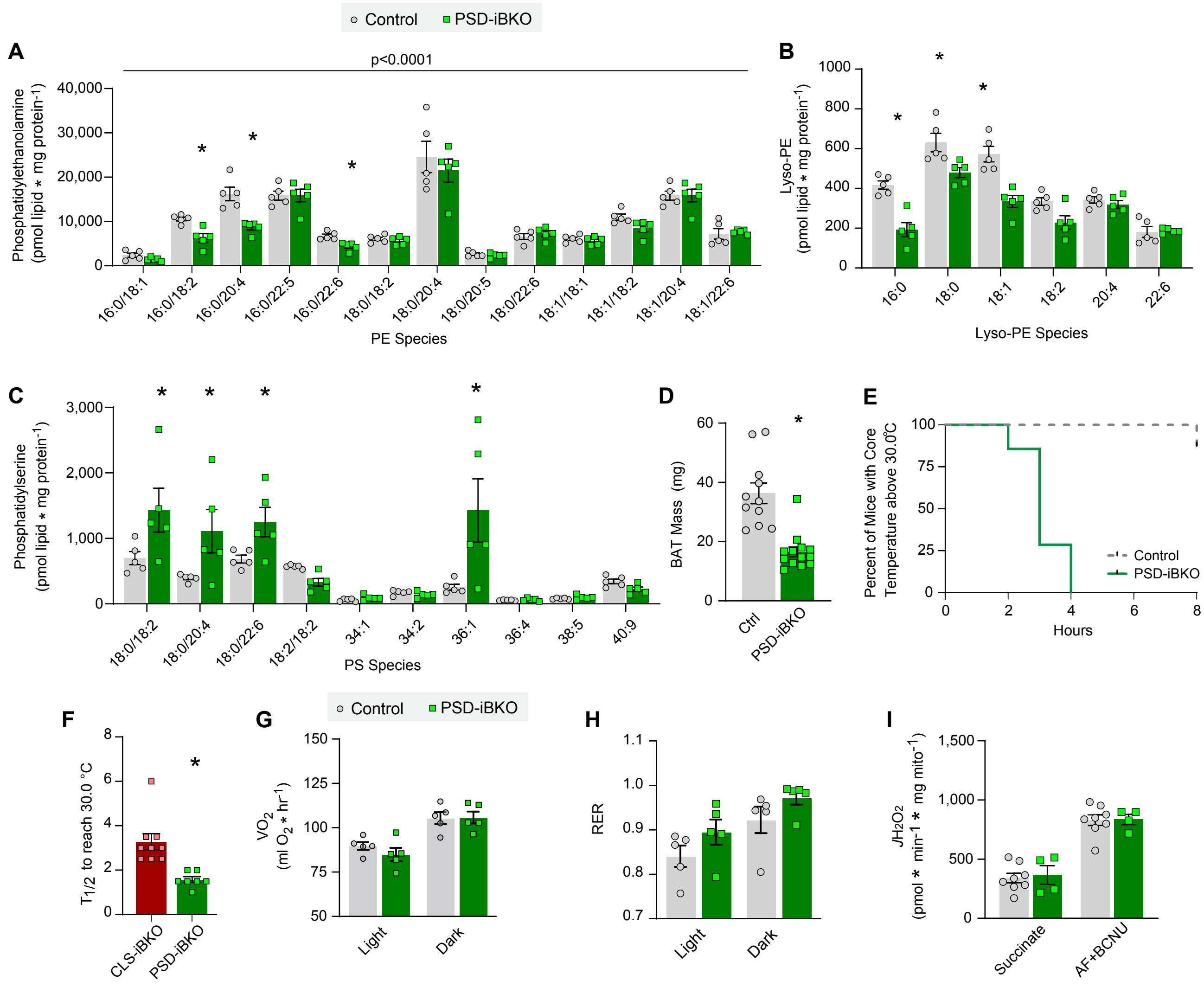

Figure S6. Additional data on PSD-iBKO mice. (**A**) Mitochondrial PE levels in BAT from control and PSD-iBKO mice, 4 weeks post-tamoxifen injection. n=5/group. (**B**) Mitochondrial lyso-PE levels, 2 weeks post-tamoxifen injection. n=5/group. (**C**) Mitochondrial PS levels, 2 weeks post-tamoxifen injection. n=5/group. (**D**) BAT mass. n=11-13/group. (**E**) Percent of mice with core body temperature above 30.0 °C during cold tolerance testing. n=7-8/group. (**F**) $T_{½}$ for core temperature to fall below 30.0 °C. n=7-9/group. (**G**) Whole-body oxygen consumption in metabolic cage. n=5/group. (**H**) Respiratory exchange ratio (RER) in metabolic cage. n=5/group. (**I**) Mitochondrial H_2O_2 production in the presence of 10 mM succinate and antioxidant inhibitors auranofin (AF) and carmustine (BCNU). n=4-8/group. Data are presented as ± S.E.M. *$p < 0.05$.

Supplemental Figure 7

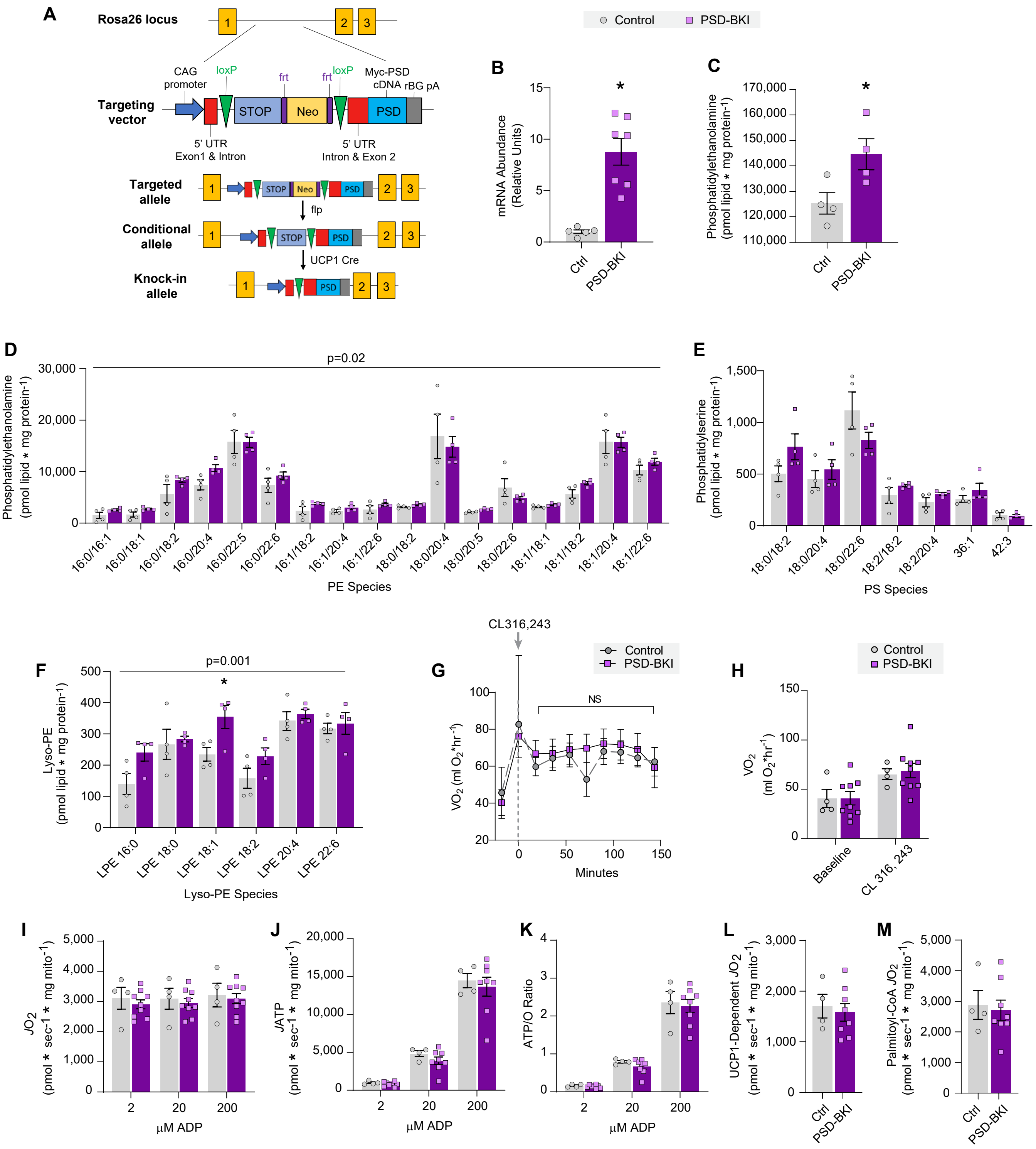

Figure S7. BAT-specific overexpression of PSD does not robustly influence mitochondrial PE, nor does it promote phenotypes in thermogenic capacity or UCP1-dependent respiration. (**A**) A schematic of the genetic strategy used to generate PSD-BKI mice. (**B**) PSD mRNA abundance in BAT from control and PSD-BKI mice. n=5-7/group. (**C**) Total mitochondrial PE in BAT from control and PSD-BKI mice. n=4/group. (**D**) Mitochondrial PE species in BAT. n=4/group. (**E**) Mitochondrial PS species in BAT. n=4/group. (**F**) Mitochondrial lyso-PE species in BAT. n=4/group. (**G**) Time course of whole-body oxygen consumption before and after CL 316,243 administration. n=4-9/group. (**H**) Mean whole-body oxygen consumption induced by CL 316,243 administration. n=4-9/group. (**I**) Mitochondrial O_2 consumption in BAT from control and PSD-BKI mice, measured in the presence of 5 mM pyruvate, 0.2 mM malate, 5 mM glutamate, and 5 mM succinate, and 2, 20, and 200 µM ADP. n=4-8/group. (**J**) ATP production in the presence of 5 mM pyruvate, 0.2 mM malate, 5 mM glutamate, 5 mM succinate, and 2, 20, and 200 µM ADP. n=4-8/group. (**K**) Mitochondrial coupling efficiency (ATP/O ratio) of BAT mitochondria. n=4-8/group. (**L**-**M**) UCP1-dependent respiration in control and PSD-BKI mice stimulated by 5 mM pyruvate and 0.2 mM malate (L) or 0.2 mM malate, 5 mM carnitine, and 20 µM palmitoyl-CoA (M) and inhibited by 4 mM GDP. n=4-8/group. All phenotyping for control and PSD-BKI were done in mice housed in thermoneutrality for 4 weeks. At room temperature housing, PSD-BKI mice did not exhibit greater mitochondrial PE content compared to control mice. Data are presented as ± S.E.M. *$p < 0.05$.
